# Mechanosensitivity of macrophage polarization: comparing small molecule leukadherin-1 to substrate stiffness

**DOI:** 10.3389/fimmu.2025.1420325

**Published:** 2025-03-06

**Authors:** Hemant Joshi, Edgar Anaya, Anvitha Addanki, Alison Almgren-Bell, Elizabeth M. Todd, Sharon Celeste Morley

**Affiliations:** ^1^ Division of Infectious Diseases, Department of Pediatrics, Washington School of Medicine in St. Louis, St. Louis, MO, United States; ^2^ Division of Immunobiology, Department of Pathology and Immunology, Washington School of Medicine in St. Louis, St. Louis, MO, United States

**Keywords:** mechanotransduction, CD11b, NLRP3, macrophages, macrophage polarization, leukadherin-1, inflammation, immune-modulation

## Abstract

Macrophages sustain tissue homeostasis through host defense and wound repair. To promote host defense, macrophages upregulate surface markers associated with antigen processing and secrete pro-inflammatory mediators such as IL-6 and IL-1β. After pathogen clearance, macrophages shift phenotype to promote wound repair. Shifts in phenotypes are termed “polarization” and have historically been modeled by exposure to soluble mediators such as LPS+IFNγ (host defense) or IL-4+IL-13 (tissue repair). Greater emphasis is now being placed on understanding how the mechanical environment of macrophages, such as tissue compliance, regulates macrophages responses. Here, we compare incubation of primary macrophages on collagen-coated silica gels of varying stiffness to treatment with the small molecule integrin activator, leukadherin-1 (LA1), to examine how substrate stiffness alters macrophage polarization in response to multiple stimuli. LA1 was developed as an immunomodulator to treat inflammatory diseases by impairing trafficking of inflammatory cells. A recent clinical trial examining LA1 as an immunomodulator in solid tumors was terminated early because no benefit was observed. We hypothesized that LA1 treatment may exert additional, unexpected effects on macrophage polarization by replicating mechanotransduction. Specifically, we hypothesized that LA1 would mimic effects of incubation on stiffer substrates, as both conditions would be predicted to activate integrins. Our results show that soft substrate (0.2 kPa) trends towards upregulation of host defense molecules, in contrast to prior reports using different experimental systems. We further show that soft substrates enhance NLRP3-mediated IL-1β production, compared to stiff, in both primary mouse and human macrophages. LA1 mimicked incubation on stiff substrates in inhibiting NLRP3 activation and in regulating expression of several surface markers but differed by reducing IL-6 production. Our results show that macrophage inflammatory responses are regulated by adhesion-based, integrin-mediated mechanical signaling. Modulation of NLRP3-mediated IL-1β production by LA1 supports the possibility of repurposing LA1 to treat NLRP3-dependent inflammatory diseases.

## 1 Introduction

Macrophages serve at least two essential functions: host defense and tissue repair. Macrophages shift metabolic, transcriptional, secretion and surface marker profiles as they balance dual roles as protectors and healers, exhibiting significant phenotypic plasticity (1–4). Several terms have historically been used to describe these profiles. “Pro-inflammatory,” or M1, indicates macrophages that have upregulated host defense genes, such as IL-6 and IL-1β, while “pro-healing,” or M2, describes macrophages secreting factors conducive to tissue repair, such as TGF-β. However, this binary categorization oversimplifies the plethora of intermediate macrophage phenotypes that simultaneously incorporate defensive and healing elements (1, 5, 6). For purposes of this article, we will use the terms “host defense” and “tissue repair” to refer to these two functions accomplished by macrophages, and “polarization” will refer to changes in any biological process than leads towards defense or repair.

Macrophages integrate multiple environmental cues to respond to tissue defense or repair needs. Environmental cues include cell surface receptors, cytokines and chemokines, available metabolites, and tissue compliance. Tissue compliance, or stiffness, changes macrophage phenotypes through a process termed mechanotransduction (7–9). Mechanotransduction is defined as the translation of external mechanical into intracellular biochemical signals that drive a biological process. Mechanical information can be translated across the macrophage cell membrane by a variety of cellular receptors, including integrins. Integrins are heterodimeric proteins that engage with surrounding extracellular matrix (ECM) and/or adhesion molecules expressed on the surface of adjacent cells. Integrins induce the formation of actin-based podosomes, organelles that recruit multiple signaling molecules to translate mechanical information into phenotypic changes (10). Integrin-mediated podosome formation also supports cell motility and migration.

Because of their critical role in cell trafficking and activation, integrins are increasingly targeted with novel immunomodulatory agents. The first-in-class CD11b agonist, leukadherin-1 (LA1), was developed as an anti-inflammatory agent that targets the leukocyte integrin Mac-1 (CD11b/CD18). By activating CD11b, LA1 increases leukocyte adhesion, preventing tissue egress and inhibiting leukocyte trafficking to sites of inflammation (11, 12). Since its development, LA1 has been used to show that CD11b agonism can reduce murine death during LPS-mediated toxic shock (13) and may reduce leukocyte trafficking in a murine model of experimental autoimmune encephalitis (14). More recently, it was proposed that the salt form of LA1 (GB1275), which is more readily orally available, could serve as an adjunctive anti-cancer therapy by reducing infiltration of macrophages and neutrophils into solid tumors (15). Unfortunately, the phase 1/2 clinical trial was terminated early because no clinical benefit to cancer patients was observed (ClinicalTrials.gov, https://clinicaltrials.gov/study/NCT04060342).

The clinical trial was sufficient to show that GB1275 is safe and well-tolerated in human patients, and it may be possible to repurpose GB1275 for use in other inflammatory disorders. Before doing so, it will be essential to fully delineate the effects of CD11b agonism on macrophages. Because LA1 activates an integrin, and because integrins mediate mechanotransduction, we propose that LA1 may induce the similar effects on macrophage differentiation, polarization and activation as does substrate stiffness. Here, we capitalize on our established model systems (16) to define the similarity of LA1 treatment and mechanosensitivity on processes such as macrophage differentiation, surface marker upregulation, and inflammasome activation, that support polarization towards host defense or tissue repair. Our results showing that macrophages cultured on softer substrates upregulate markers traditionally associated with host defense, in contrast to many prior reports, suggesting that mechanical regulation is more complex than previously appreciated. We further show that treatment with LA1 also alters key macrophage co-receptors, such as CD206 and CD74. Finally, we show that NLRP3 inflammasome activation is downregulated by both LA1 treatment and incubation on stiffer substrates. Our findings show that integrin agonism, either through LA1 or via engagement with stiff substrates, are a key factor in mediating macrophage polarization.

## 2 Materials and methods

### 2.1 Experimental model and subject detail

All animal procedures are conducted in accordance with the National Institute of Health (NIH) Guide for the care and Use of Laboratory animals and approved by the Institutional Animal Care and Use Committee (IACUC), Washington University School of Medicine in St. Louis (WUSM), St. Louis, MO, USA. Animals were housed in specific-pathogen free barrier facilities and were routinely monitored for general health, cage changes, feeding and overcrowding. Mice were matched for age (8-10 weeks) and biological sex. All mice were C57BL/6 background.

### 2.2 Macrophage culture and differentiation

For bone marrow derived macrophages (BMDMs), mice femurs and tibias were harvested, and bone marrow was flushed with DPBS containing 1% FBS. Collected bone marrow cells were treated with BD RBC lysis buffer (BD; Cat#555899). To induce differentiation to macrophages, bone marrow cells were cultured in DMEM supplemented with 10% L929 conditioned media (containing macrophage colony stimulating factor (M-CSF) and 10% FBS, 1% penicillin/streptomycin (Lonza; Cat# BW17602E). BMDMs were collected using Cellstripper™ buffer (Corning; Cat#25-056-CI) after 7 days of culture and then further used for stimulations.

Human monocyte derived macrophages (HMDMs) were differentiated from packs of peripheral blood mononuclear cells (PBMCs) obtained from anonymized healthy donors by a blood bank. Because donations are anonymized and no personal health information is included, this process is IRB exempt. For differentiation, PBMCs were cultured in presence of M-CSF (100 ng/ml) (Gibco: Cat.# PHC9504) for 7 days (fresh media containing M-CSF was added on day 3).

### 2.3 *In vitro* macrophage stimulation

#### 2.3.1 Induction of macrophage polarization

BMDMs were cultured for 24 h with LPS (100 ng/ml) plus IFNγ (50 ng/ml) (R&D systems Cat.# 485-MI-100/CF) for classical activation (host defense) and with IL-4 (Abcam cat.# ab259406) plus IL-13 (BD biosciences B554599 (both 20ng/ml) for alternative activation (tissue repair).

#### 2.3.2 Inflammasome activation

For *in vitro* activation, BMDMs, and HMDMs were maintained in DMEM supplemented with 10% fetal bovine serum (FBS) (17). For NLRP3 activation, priming was provided using LPS (500 ng/ml for BMDMs) (InvivoGen; Cat#tlrl-3pelps) for 4 hrs. After that, specific NLRP3 inflammasome activators nigericin (20µM; Sigma Aldrich; Cat#N7143; 30 min) or ATP (5mM; Sigma (Roche); Cat#10127523001; 30 m) were added, as indicated. Exceptions with other concentrations or durations are specified in the figure legends. For NLRC4 inflammasome activation, macrophages primed with LPS (500 ng/ml for 4 h) were transfected with purified flagellin (1 µg/ml for 1 h; S. typhimurium; InvivoGen; Cat#tlrl-stfla) using DOTAP liposomal transfection reagent (Sigma (Roche); Cat#11202375001) for 2 h in OptiMEM media (gibco; Cat#31985070) (Miao et al., 2006).

To test mechanotransduction, macrophages were cultured on Collagen-1 (Corning; Cat#354236, 100µg/ml) coated silica gel containing glass bottom 96 well plates (Advanced BioMatrix; Cat#5255 and #5261). For engaging CD11b integrin receptor, specific activator Leukadherin-1 (LA-1 (ADH-503 (GB1275)) from Selleck Chemicals (Cat.#SO525) was administrated in culture 30 min before inflammasome activation (during the last 30 min of LPS-mediated priming. Prior experiments examining integrin-mediated podosome formation in BMDMs plated on plastic have shown that integrins can be effectively stimulated when BMDMs are engaged with a stiff surface (18). We chose to test the effect of LA-1 treatment while BMDMS were plated on plastic to follow standard cell culture procedure and to ensure comparison to a majority of other studies using cultured BMDMs.

### 2.4 ELISA

Culture supernatants (fresh or stored at -80°C) from stimulated macrophages were determined by using ELISA detection kits to measure secreted mouse IL-1β (AB_2574946) or human IL-1β (Invitrogen; Cat# 88-7261-22) as per manufacturer instructions. In parallel, for LPS priming signal, mouse TNF-α (AB_2575080), mouse IL-6 (AB_2574989), or human IL-6 (Invitrogen; Cat#88-7066-22) were detected from culture medium using ELISA technique as per manufacturer instructions. IL-1β quantification was normalized to corresponding levels of IL-6, to standardize for priming efficiency across multiple experiments.

### 2.5 Microscopy

#### 2.5.1 Macrophage circularity

BMDMs from WT mice were plated on collagen coated 0.2 kPa and 64 kPa stiffness gels in glass bottom plates. After 24 h of incubation, images were obtained using Nikon TsR2 epifluorescence illumination microscope with 20× air objective, 0.75 numerical aperture. Area of cells was determined from captured images using FIJI (ImageJ).

#### 2.5.2 Analysis of phospho-Yap1 and Yap1

BMDMs were seeded at 5x10^4^ well in 8 chambered glass slides (Merck Millipore Sigma; cat# PEZGS0816). Inflammasome activation was performed as described above. Cells were fixed with 4% paraformaldehyde (Electron Microscopy Sciences; cat# 15710S), permeabilized with 0.25% Triton x-100, blocked in 5% normal goat serum for 30 min, then stained with anti-phosphoYap1 (S127; ThermoFisher; cat# PA5-17481); anti-Yap1 (63.7; Santa Cruz Biotechnology; cat# sc-10199) at 1:75 incubated overnight. Anti-pYap1 antibody was detected via secondary mouse antibody conjugated to DyLight-594 at 1:100 (Jackson ImmunoResearch Laboratories, West Grove, PA). Anti-Yap1 antibody secondary was Goat Anti-Mouse IgG H&L-Alexa Fluor 647 at 1:200 (abcam; cat# ab150115). Phalloidin-AlexaFluor-488 (Invitrogen; cat#A12379) was used to stain for F-actin. Nuclei were visualized with DAPI 1:1000 (Sigma; cat#32670).

Images for pYap1 were acquired using a Nikon Spinning Disk Confocal Microscope with Hamamatsu Camera (C14440-20UP SN:000409) with a 60x oil objective and numerical aperture 1.40, all equipment provided by The Washington University for Cellular Imaging (WUCCI).

Images for Yap1 are captured from Zeiss LSM880 with a 40x oil objective and numerical aperture of 1.4, microscope is provided by the Molecular Microbiology Imaging Facility at Washington University School of Medicine

Analysis was conducted using FIJI (ImageJ) to generate a z-projection that averaged the pixels from the stack; region of interest for the whole cell was outlined using phalloidin and then measured total fluorescence of anti-pYap1 and Yap1. Nuclei were outlined using DAPI channel, then nuclear fluorescence of anti-pYap1 or Yap1 quantified. Once measurements where obtained they were calculated by the following equation; corrected total cell fluorescence (CTCF) = Integrated Density – (Area of selected cell X Mean fluorescence of background readings), (Measuring Cell Fluorescence Using ImageJ, 2014). Cytoplasmic intensity of pYap1 or Yap1 was determined for each cell by subtracting nuclear intensity from total cell intensity. Data from 20-30 randomly selected cells were collected from each experimental group.

### 2.6 Flow cytometry

BMDMs were harvested after stimulation and stained with directly fluorescently labeled antibodies used at 1:200 dilution as per manufacturer’s instructions. Flow cytometry analysis was performed at BD Fortessa LSRII (BD Bioscience), and data was analyzed using FlowJo (BD) software. The initial flow cytometric analysis to exclude dead cells, debris and singlets and to confirm identity as BMDMs (CD11b^+^ and F4/80^high^) is shown in **Supplementary Figure 1**.

Fluorescently-labeled, validated monoclonal antibodies were commercially obtained: CD11b- PE-Cy7 (AB_312799), CD45- BV510 (AB_2563378), F4/80-APC (AB_893493), Ly6G-PE (AB_893493), Ly6C-PercP-Cy5.5 (AB_1659242), MHCII-BUV395 (BD Bioscience Cat.#569244), SiglecF-AF647 (AB_2687570), CD40- Pacific Blue™ (BioLegend Cat.# 124625), Ly6C-BV605 (BioLegend Cat.# 128036), Ly6G-BV421 (BioLegend Cat.# 127628), CD74-Alexa Fluor^®^ 647 (BioLegend Cat.# 151004), CD64-PE (BioLegend Cat.# 139304), CD36-Alexa Fluor^®^ 488 (BioLegend Cat.# 102607), CD80- PE/Fire (BioLegend Cat.# 104759), CD86-PE (BioLegend Cat.#105008) CD9- PerCP-Cy5.5 (BioLegend Cat.# 124817), CD209a- PE (BioLegend Cat.# 833004), CD31- Biotin ((BioLegend Cat.# 102404), CD206- PE-Cy7 (BioLegend Cat.# 141719), iNOS-PE-Cy7 (ThermoFisher Cat.# 25-3920-80), Arginase-1-APC (Life Technologies Cat.# 17-3697-80).

### 2.7 Immunoblotting

Cell lysates were prepared using RIPA lysis buffer containing protease and phosphatase Inhibitors (Sigma Aldrich: Cat#PPC1010) and further processed in Laemmli buffer and stored in -20°C. Cell lysates were subjected to SDS-PAGE and resolved protein bands were transferred on PVDF-membranes (Bio-Rad; Cat#165800). For detecting proteins, membranes were incubated with specific primary antibodies overnight at 4°C. Primary antibodies diluted (1:1000) in TBST with 5% milk or 3% BSA. Secondary antibody directed to primary antibody used at dilution of 1:10000; anti-rabbit IgG-AF680, anti-Rabbit IgG DyLight800, anti-mouse IgG-IRDye800, anti-mouse IgG-AF680. For detecting IL-1β, supernatants were incubated with StrataClean resin (Agilent Technologies; Cat#400714) overnight. Beads bound proteins were eluted by boiling with Laemmli buffer and used for immunoblotting. For imaging, membranes were scanned in LI-COR- Odyssey imager.

To ensure equal protein loading from cell lysates, aliquots from each sample were “pre-run” for immunoblot for β-actin. Densities of β-actin bands were then used to normalize equivalent protein loading for samples on subsequent immunoblots. Membranes were cut along molecular weight markers and incubated in different primary antibodies so that multiple proteins could be quantified from the same experiment without stripping and reprobing. Additionally, secondary antibodies were conjugated with infra-red dyes and imaged using the LiCOR Odyssey system, which permits identification of two separate proteins at similar molecular weights (e.g. phospho-ERK1/2 and total ERK1/2) without stripping and reprobing. Densitometry analysis was performed using ImageStudioTM (LI-COR) software. Primary and secondary antibodies are below.

NLRP3 (AB_2722591), IL-1β (AB_416684), Caspase-1 (AB_2068894), Cleaved Caspase-1 (Cell Signaling Tech. Cat.# 89332S), gasdermin (Cell Signaling Tech. Cat #97558t) β-Actin (Cell Signaling Tech. Cat.#4970s), phosphoERK2 (Cell Signaling Tech. Cat.#4370s), phosphoJNK1 (Invitrogen Cat./# PA5-37698), phosphoNF-kB (Cell Signaling Tech. Cat.# 30), phosphoYap1 (Invitrogen Cat.# PA5-17481), phosphoP38 (Cell Signaling Tech. Cat.#4511S), ERK2 (Cell Signaling Tech. Cat.#4696), JNK (OriGene Cat.#TA13291S), NF-kB (Novus Cat.# NBP2-27416SS), Pyk2 (Cell Signaling Tech. Cat.#3480S), P38 (Cell Signaling Tech. Cat.#9217), Anti-Rabbit-IgG-AF680 (AB_2535758), anti-Rabbit IgG DyLight™ 800 (AB_2610841), anti-goat IgG IRDye800, anti-mouse IgG-IRDye800 (AB_220125).

### 2.8 Quantification and statistical analysis

Immunoblots were visualized using a LI-COR Odyssey imager. Densitometry analysis was performed using ImageStudio™ (LI-COR) software. Microscopic images were analyzed using ImageJ software (NIH). Flow cytometry results calculated (frequency and median fluorescent intensity (MFI)) by FLOWJO (BD) software. ELISA results were quantified using spectrophotometer at 450 nm absorbance (BioTek).

Quantified data were graphed and analyzed using Prism (GraphPad Software, La Jolla, CA). Violin plots show distribution of data with medians indicated. Non-parametric Mann-Whitney, Wilcoxon or Kruskal-Wallis (with follow-up for multiple comparisons) tests were employed, as indicated in figure legends. P-values ≤ 0.05 were considered significant.

## 3 Results

### 3.1 Experimental design

**Figure 1:**
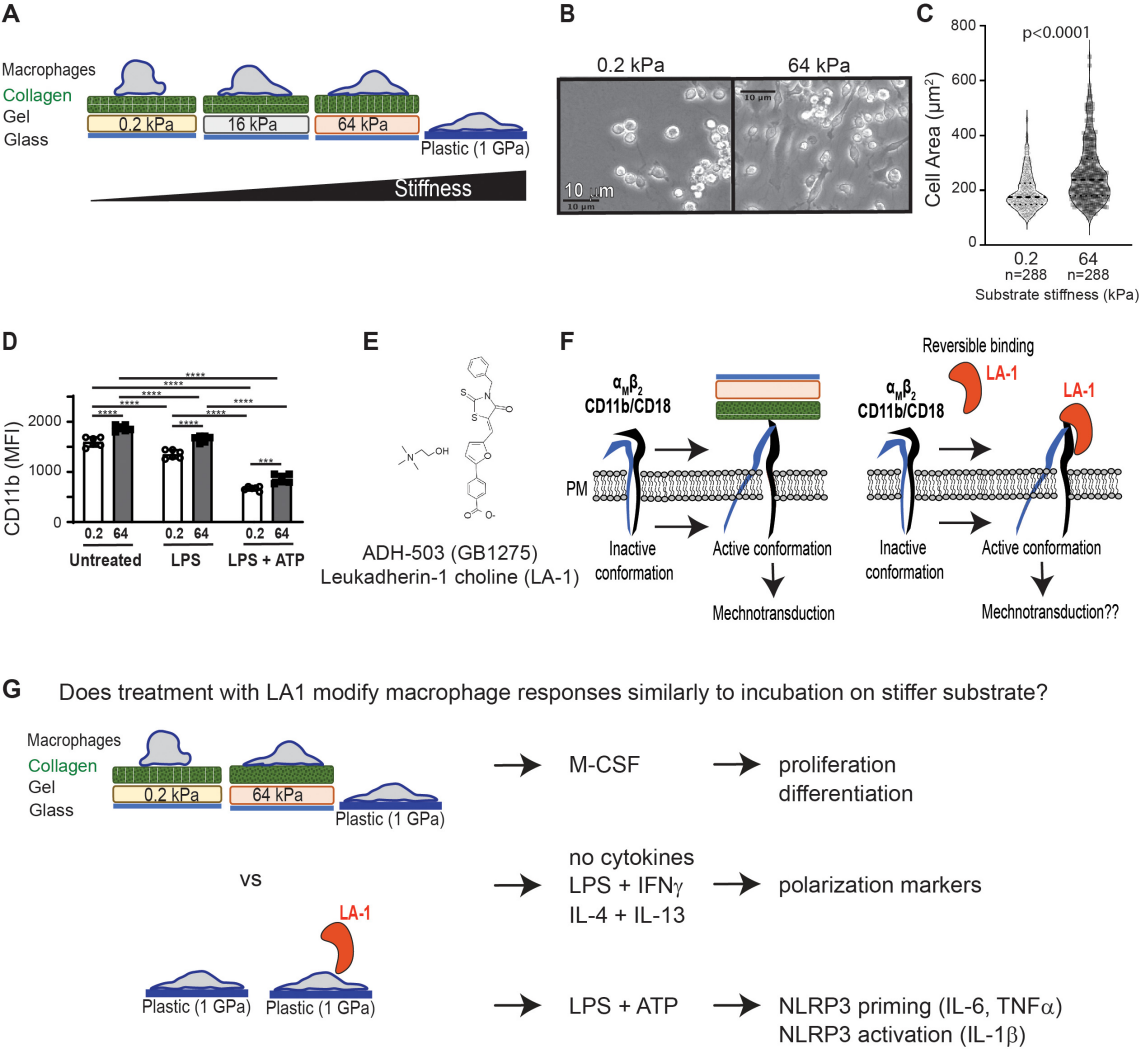
Summary of experimental systems and plan. **(A)** Schematic depiction of *in vitro* cell culture model to test mechanotransduction by incubation of murine BMDMs on collagen-coated silica gels with corresponding stiffness (quantified as kilopascal, kPa) **(B)** Brightfield images from BMDMs cultured on gels of 0.2 kPa (soft) or 64 kPa (stiff). Scale bar = 10 µm. **(C)** Area of BMDMs measured from microscopic images shown in **(B)**. Each symbol represents area of one cell, line at median, p-value determined by Mann-Whitney, data from three independent experiments. **(D)** Flow cytometric analysis of CD11b expression on BMDMs cultured on gels of indicated stiffness and primed with LPS, primed with LPS and activated with ATP, or left unstimulated. Each symbol represents value from one sample, bar at median, p-values determined by two-way ANOVA indicated significant effect of mechanosensation (MS) and cytokine stimulation (CS), with follow-up pairwise comparisons (***p< 0.001, ****p< 0.0001). Data from 3 independent experiments. **(E)** Molecular structure of CD11b activator LA-1 (12). **(F)** Schematic showing hypothesized activation of integrins on stiff substrate to induce mechanotransduction, compared to LA-1 ligation and activation of CD11b. **(G)** Summary of experimental plan to test if exposure to LA-1 and incubation on stiffer substrates similarly regulate BMDM activation and polarization.

We have established an *in vitro* model system for inducing mechanotransduction in bone marrow-derived cells (**Figures 1A–C**). In brief, bone marrow-derived monocytes or macrophages (BMDMs) are incubated on collagen-coated silica gels or on untreated tissue culture plates. Gel compliance is selected to mimic *in vivo* tissue compliance, which ranges from 0.2 kPa (brain, healthy lung) to 64 kPa (fibrotic tissue, cartilage) (19). Standard, non-tissue culture (TC)-treated plastic dishes are included as the control substrate (about 1 GPa, or 1 x 106 kPa) (20) and represent standard *in vitro* TC practices (**Figure 1A**). Mechanotransduction on collagen-coated gel substrates is confirmed by greater spreading of BMDMs on stiffer substrates, while BMDMs remain relatively round on soft substrate (**Figures 1B, C**). Quantification of the area of BMDMs after culturing on soft (0.2 kPa) or stiff (64 kPa) substrates after 24 h shows increased spreading of BMDMs on stiffer substrates, validating mechanotransduction in our *in vitro* system (**Figure 1C**).

Mechanotransduction is enabled in part by integrins (19), such as CD11b/CD18. CD11b is expressed on macrophages, is mechanosensitive, and is maintained after inflammatory stimulation (**Figure 1D**). CD11b can be directly activated by the small molecule leukadherin-1 (bioavailable salt form, GB1275, shown; **Figure 1E**). We hypothesized that direct agonism of CD11b would mimic incubation on stiffer substrates, because incubation on stiffer substrates engages and activates integrins (**Figure 1F**). To test our hypothesis, we compared M-CSF-induced proliferation and differentiation, phenotypic changes in common polarization markers, and NLRP3 inflammasome activation in BMDMs either cultured on gels of varying compliance or treated with LA1 (**Figure 1G**). Previous work on podosome activation has shown that integrins can be activated when cells are incubated on tissue culture plates (18), and to enable comparison of our results to prior publications, we elected to expose macrophages to LA1 using standard tissue culture procedures (i.e. incubated in plastic plates). In some instances, we further extended our analysis of mechanotransduction and macrophage polarization to human monocyte-derived macrophages (MDMs).

### 3.2 Mechanosensitivity of differentiation from monocyte to macrophage

**Figure 2:**
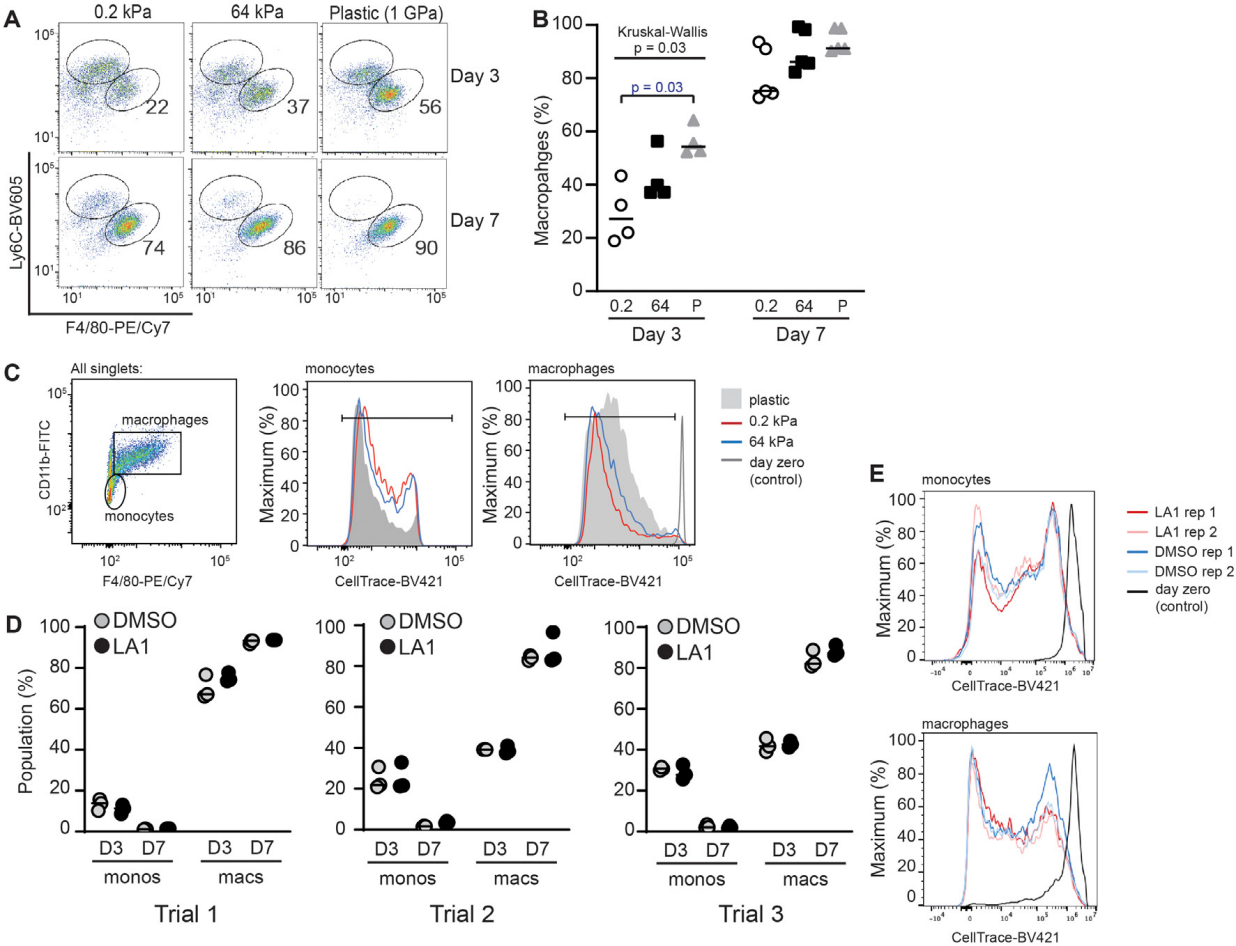
Stiffness of collagen-coated silica gels, but not LA1 treatment, regulates BMDM differentiation. **(A)** Bone-marrow cells from WT mice were incubated on collagen-coated silica gels or TC plastic in M-CSF (L929-cells supernatant) for three or seven days. Cells were analyzed by flow cytometry for Ly6C and F4/80 to identify populations of monocytes (Ly6C^high^F4/80^low^) and BMDMs (Ly6C^low^F4/80^high^). Percentage of BMDMs indicated in each flow plot. **(B)** Percentage of total cells that were identified as BMDMs. Each symbol represents value from one of three independent experiments, line at median, Kruskal-Wallis with follow-up pairwise comparison used to determine p-values, “P” = plastic. **(C)** Monocytes were labeled with CellTrace-BV421 at start of incubation. After 3 days in culture, dilution of CellTrace-BV421 was analyzed by flow cytometry. Cells were defined as monocytes or macrophages as shown. **(D)** Bone marrow cells from WT mice were labeled with CellTrace-BV421, then incubated with or without LA1 (5 μg/ml) for 3 or 7 days. Flow cytometric analysis was used to determine surface expression of CD11b, Ly6C, and F4/80 and dilution of CellTrace-BV421. Monocytes and BMDMs were determined as in **(A)**. Each symbol shows value for one sample, with each experiment performed with technical triplicates. Line at median. Results of three independent experiments are shown. **(E)** Dilution of CellTrace-BV421 dilution from cells in **(D)**. Representative samples from one of three independent experiments shown.

During inflammation, peripheral blood monocytes traffic into target organs, then differentiate into inflammatory macrophages. Historically, blood monocytes have been regarded as non-proliferating, with increased bone marrow production and recruitment driving increased populations of inflammatory monocytes in target organs (21, 22). However, recent studies challenge this paradigm, as monocytes may initially proliferate in empty niches to rapidly repopulate tissue macrophages (23, 24). The external cues guiding differentiation of monocytes into macrophages, and possible proliferation of monocyte and macrophage populations, in inflamed organs are not fully defined, and may include mechanotransduction (25). We postulated that changing substrate stiffness might alter the proliferative capacity of monocytes. We therefore compared *in vitro* proliferation of monocytes and differentiated BMDMs and differentiation of bone marrow-derived monocytes into BMDMs on stiff (64 kPa) and soft (0.2 kPa) substrates (**Figures 2A–C**). Monocytes were defined as Ly6C^high^F4/80^neg^, while macrophages were defined as Ly6C^low^F4/80^high^. After 3 days of incubation, we observed a significantly greater proportion of differentiated BMDMs on stiff substrates. After 7 days in culture, almost all cells were differentiated into macrophages (**Figure 2B**). We interpret these data to indicate that stiffer substrate induces earlier commitment of monocytes to macrophage differentiation, but that soft substrates do not inhibit eventual macrophage differentiation.

We measured proliferation by labeling monocytes with CellTrace-BV421 after isolation from the bone marrow, before initiating *in vitro* culture. Proliferation was visualized by CellTrace-BV421 dilution over time. Monocytes and macrophages were defined as F4/80^neg^ or F4/80^pos^, respectively, and proliferation of each population quantified (**Figure 2C**). Dilution of CellTrace-BV421 appeared equivalent in monocyte and macrophage populations incubated on 0.2 kPa and 64 kPa substrates, indicating that proliferation was not mechanosensitive in our system (**Figure 2C**). The possible difference in CellTrace-BV421 dilution between cells cultured on plastic plates and those incubated on silica gels was not a reproducible finding. We note that both monocyte and macrophage populations demonstrated CellTrace-BV421 dilution, indicating that both populations exhibit proliferative capacity.

We then compared the effects of LA1 treatment of monocytes on the first day of incubation to those of incubating on substrates of varying compliance. Monocytes were labeled with CellTrace-BV421, incubated with or without LA1 in plastic plates for 72 h or 7 d, then analyzed by flow cytometry as in **Figures 2A,C** (**Figures 2D,E**). The percentages of differentiated macrophages and remaining monocytes were similar in LA1-treated and in untreated conditions across three independent trials (**Figure 2D**). We also observed no difference in CellTrace-BV421 dilution with or without LA1 (**Figure 2E**). Thus, we found evidence that monocyte to macrophage differentiation varies with substrate stiffness, but that LA1 treatment does not replicate mechanosensitivity induced by substrate stiffness. *In vitro* proliferation did not appear to be sensitive to substrate stiffness or to LA1 treatment in our system.

### 3.3 Mechanotransduction and cytokine stimulation co-regulate markers associated with pro-defense and pro-healing functions

**Figure 3:**
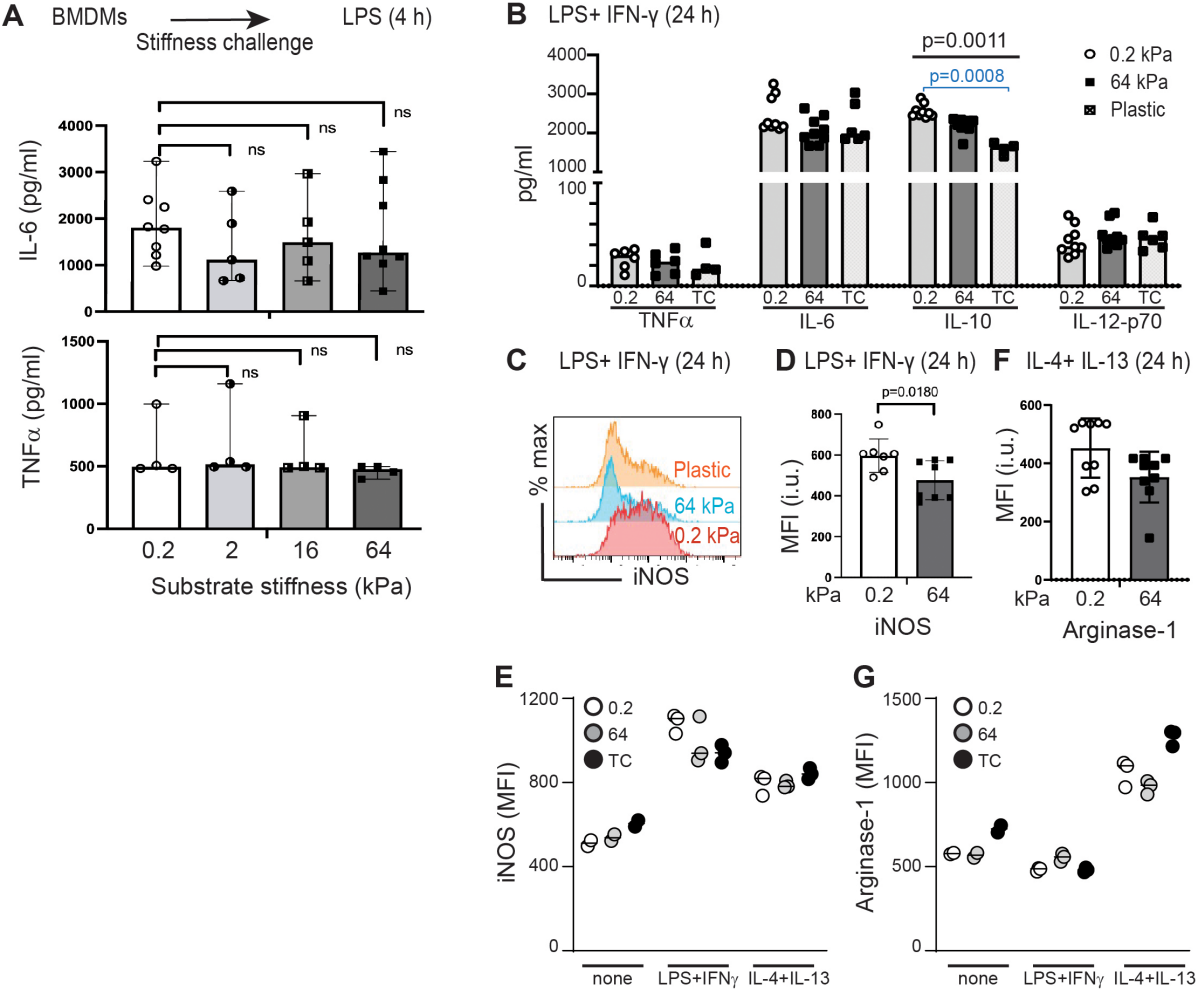
Testing the effects of substrate stiffness on induction of selected cytokines, iNOS, and arginase-1. **(A)** IL-6 and TNFα production from BMDMs cultured on collagen-coated gels of varying stiffness for 24 h prior to short-term (4 h) LPS (0.5 µg/ml) stimulation. Culture supernatants were analyzed by ELISA. Each symbol represents value from an independent sample; bar indicates median value, ranges shown. Mann-Whitney was used to compare two groups. **(B)** TNFα, IL-6, IL-10, and IL-12p70 production from BMDMs incubated on collagen-coated gels of varying stiffness and stimulated (24 h) with LPS+IFNγ. Culture supernatants were analyzed by ELISA. Each symbol represents value from an independent sample; bar indicates median value. ANOVA used to compare three groups with follow-up pairwise comparison. Only IL-10 production showed significant differences across three groups (ANOVA p = 0.0011), with follow-up pairwise comparison showing a significant difference between 0.2 kPa and plastic (p = 0.0008). **(C)** Flow cytometric analysis of iNOS expression in BMDMs cultured collagen-coated gels of varying stiffness and stimulated (24 h) with LPS+IFNγ. **(D)** MFI of iNOS from BMDMs cultured as in **(C)**. Each symbol represents value from an independent sample; bar indicates median value, interquartile ranges shown, p-value determined using Mann-Whtiney. **(E)** MFI of iNOS from BMDMs cultured on collagen-coated gels of varying stiffness and stimulated with LPS+IFNγ, IL-4+IL-13, or media only. Each symbol represents value from one sample, line at median. Data from one of three independent experiments, with technical triplicates shown. No statistical tests performed as n=3 too small. **(F)** MFI of arginase-1 expression in BMDMs cultured on collagen-coated gels and stimulated (24 h) with IL-4+IL-13. Each symbol represents value from an independent sample; bar indicates median value, interquartile ranges shown, p-value determined using Mann-Whtiney. **(G)** MFI of arginase-1 from BMDMs cultured on collagen-coated gels of varying stiffness and stimulated with LPS+IFNγ, IL-4+IL-13, or media only. Each symbol represents value from one sample, line at median. Data from one of three independent experiments, with technical triplicates shown. No statistical tests performed as n=3 too small. Data from **(E)** and **(G)** are included in **(D)** and **(F)**, respectively.

While multiple studies have shown that substrate stiffness polarizes monocytes and macrophages, how stiffness interacts in combination with stimulatory cytokines to co-regulate polarization is not yet fully defined (19, 25–29). We first measured concentrations of IL-6 and TNFα secreted by BMDMs after 4 h of LPS-stimulation while incubated upon collagen-coated silica gels of varying stiffness (**Figure 3A**). Under conditions of short stimulation, we did not find evidence of mechanosensitivity of cytokine secretion. We then tested if longer exposure (24 h) to the pro-inflammatory combination of LPS+IFNγ would reveal mechanosensitivity of cytokine expression. Secretion of IL-10 was increased in BMDMs incubated upon softer substrates (**Figure 3B**), but we did not find significant changes in concentrations of TNFα, IL-6 or IL-12p70 (**Figure 3B**). BMDMs incubated upon collagen-coated silica gels and either exposed to IL-4+IL-13 (24 h) or left without specific cytokine stimulation did not produce significant amounts of TNFα, IL-6, IL-10 or IL-12p70 (data not shown).

Production of nitric oxygen species, measured as intracellular inducible nitric oxygen synthase (iNOS) is induced by the pro-inflammatory combination of LPS+IFNγ, while production of arginase-1 is induced by the pro-healing cytokine combination of IL-4+IL-13. Incubation of BMDMs with LPS+IFNγ on soft substrate resulted in significantly greater production of iNOS, indicating mechanosensitivity of iNOS production (**Figures 3C,D**). Control samples reveal the relative expression of iNOS in macrophages exposed to LPS+IFNγ, IL-4+IL-13, or assessed without specific cytokine stimulation (**Figure 3E**). We did not find that incubation on stiff or soft substrates induced significant differences in arginase-1 upregulation after incubation with IL-4+IL-13 (**Figure 3F**), although control comparisons confirmed that IL-4+IL-13 induced Arg-1 above baseline and above incubation with LPS+IFNγ (**Figure 3G**).

**Figure 4:**
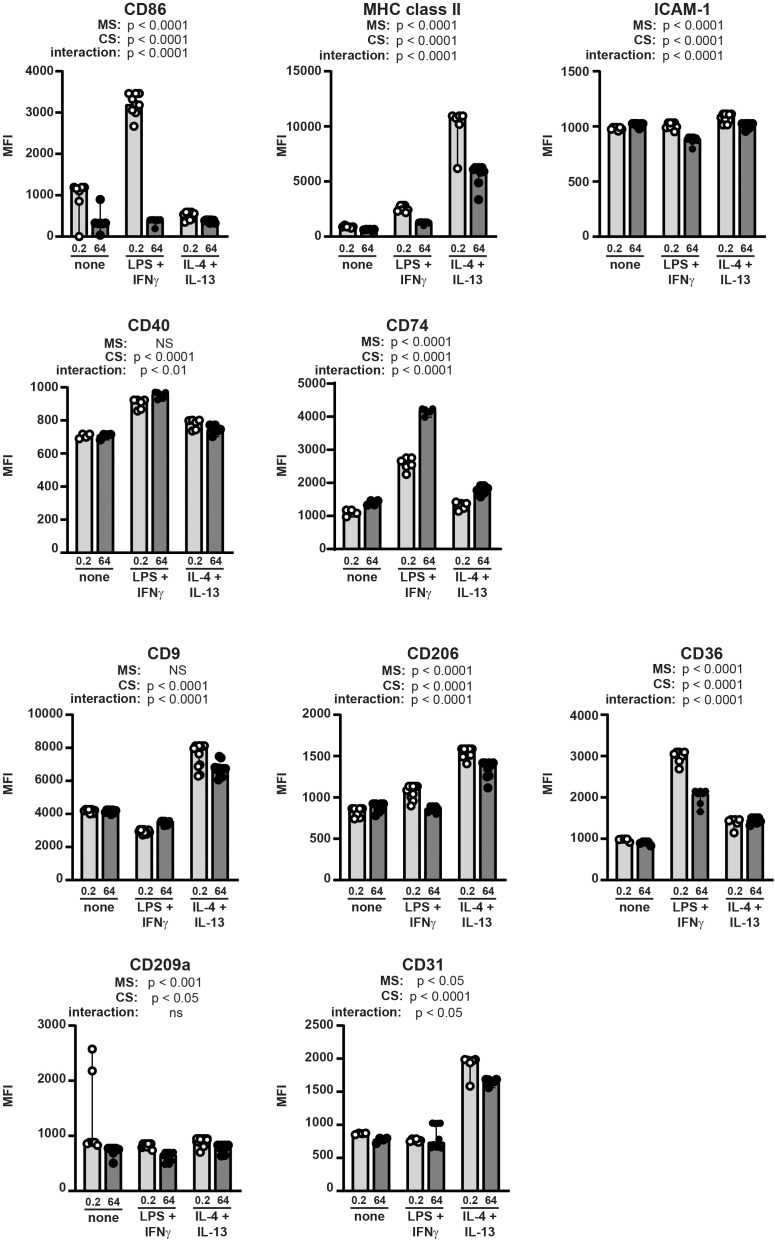
Macrophage surface markers are differentially regulated by integration of mechanical and cytokine cues. BMDMs were incubated on collagen-coated silica gels (open circles, light grey bars: 0.2 kPa; closed circles, dark grey bars: 64 kPa) with LPS+IFNγ, IL-4+IL-13, or without specific cytokine stimulation (none) for 24 h. Expression of indicated surface markers were analyzed by flow cytometry and quantified as median fluorescence intensity (MFI) of all BMDMs gated (CD11b^pos^F4/80^pos^). Each symbol represents value from one sample; bar shows median value; 95% confidence interval shown. Data were combined from three independent experiments, enabled by consistent fluorophore staining and identical cytometer settings for independent acquisitions. Two-way ANOVA was used to determine if substrate stiffness (MS, mechanosensation) and/or cytokine stimulation (CS) significantly altered marker expression, and to determine if there was a significant interaction of mechanosensation and cytokine stimulation on MFI. No specific pairwise comparisons were tested.

We then tested the mechanosensitivity of surface marker expression by BMDMs incubated on stiff and soft substrates without additional stimulation, with LPS+IFNγ, or with IL4+IL-13 (**Figure 4**). We selected surface markers to analyze by flow cytometry that have been previously reported to be associated with macrophage polarization (30). Host defense phenotypes have been previously correlated with upregulated CD86, MHC class II, ICAM-1, CD40, and CD74, while tissue repair has been associated with upregulated CD9 and CD206 (30). CD36 and CD209a have been previously shown to be mechanosensitive (31, 32). Finally, CD31 was selected as a control because it is an integrin and increased CD31 expression has been previously associated with some inflammatory conditions (33). Expression of selected markers was quantified as median fluorescent intensity (MFI) of marker on all BMDMs analyzed.

Expression of all surface markers analyzed was significantly altered by substrate stiffness, cytokine exposure, or both (**Figure 4**). We analyzed statistical significance of the expression levels of each marker expressed by BMDMs on stiff or soft substrate in the presence or absence of polarizing cytokines using a two-way ANOVA (MS: mechanosensitivity; CS: cytokine stimulation). The two-way ANOVA tests if each of two separate independent variables affect one dependent variable, and tests if the two independent variables interact with each other. We thus are comparing all six conditions in one statistical test (no pairwise comparisons), and analysis results are provided underneath each marker label. For instance, CD86 expression was upregulated by LPS+IFNγ on soft substrate, but not on stiff, indicating significant regulation by cytokine stimulation and by substrate stiffness, and revealing that a significant interaction between substrate compliance and cytokine exposure. MHC class II expression was higher on soft substrate compared to stiff after exposure to either LPS+IFNγ or IL-4+IL-13. ICAM-1 was downregulated after cytokine exposure on stiff substrate, but not soft. CD40 expression changed significantly with cytokine stimulation but was not altered by substrate stiffness. CD74 was higher on stiff substrate, with greatest expression after exposure to LPS+IFNγ.

CD9 was greatly upregulated by IL-4+IL-13 but was not mechanosensitive. CD206 was also expressed most highly after IL-4+IL-13 and was mechanosensitive (higher on soft substrates). CD36 expression was regulated similarly to CD86 in that expression was highest on soft substrate after exposure to LPS+IFNγ. CD209a was regulated primarily by mechanosensitivity (higher on soft substrate) with some contribution from the cytokine milieu, while CD31 was greatly upregulated by exposure to IL-4+IL-13 and only mildly mechanosensitive.

**Figure 5:**
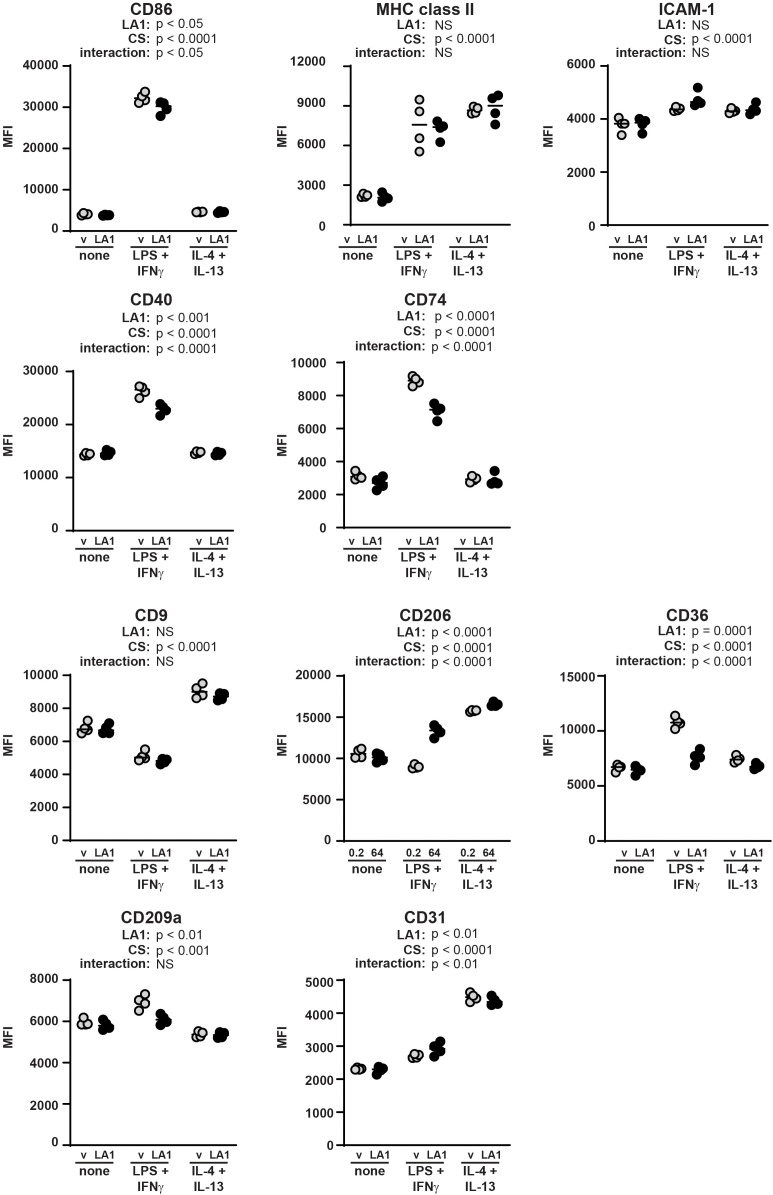
Macrophage surface markers are differentially regulated by LA1 treatment and cytokine cues. BMDMs were incubated with (closed circles) or without (gray circles) LA1 (5 µg/ml) and with LPS+IFNγ, IL-4+IL-13, or without specific cytokine stimulation (none) for 24 h. Expression of indicated surface markers were analyzed by flow cytometry and quantified as median fluorescence intensity (MFI) of all BMDMs gated (CD11b^pos^F4/80^pos^). Each symbol represents value from one sample; bar shows median value. Two-way ANOVA was used to determine if LA1 exposure (LA1) and/or cytokine stimulation (CS) significantly altered marker expression, and to determine if there was a significant interaction of LA1 exposure and cytokine stimulation on MFI. No specific pairwise comparisons were tested. Results from one representative experiment of two or three independent experiments shown; variability across independent acquisitions precluded combining data from multiple experiments. Results from other experiments are shown in **Supplementary Figure 2**.

We then analyzed the same surface markers on BMDMs incubated in polarizing cytokines with or without LA1 (**Figure 5**; **Supplementary Figures 1, 2**). All markers demonstrated similar responsiveness to polarizing cytokines as observed in **Figure 4**. Incubation with LA1 exerted similar effects on CD36, CD209a, and CD31 as did incubation on stiff substrates. LPS+IFNγ-mediated upregulation of CD86 was mildly reduced by LA1 treatment, compared to the profound reduction on stiffer substrates. Incubation with LA1 exerted significant but opposite effects on CD74 and CD206 surface expression, as compared to incubation on stiff substrates. CD9, MHC class II and ICAM-1 were not significantly impacted by LA1 exposure. Upregulation of CD40 by LPS+IFNγ was reduced by LA1, although we saw no effect of substrate stiffness on CD40 expression. Thus, specific activation of CD11b by LA1 exerted similar effects as stiff substrates on a subset, but not all, surface markers associated with macrophage polarization (**Figure 5**; **Supplementary Figure 2**).

### 3.4 NLRP3-mediated production of the host defense cytokine IL-1β is mechanosensitive

**Figure 6:**
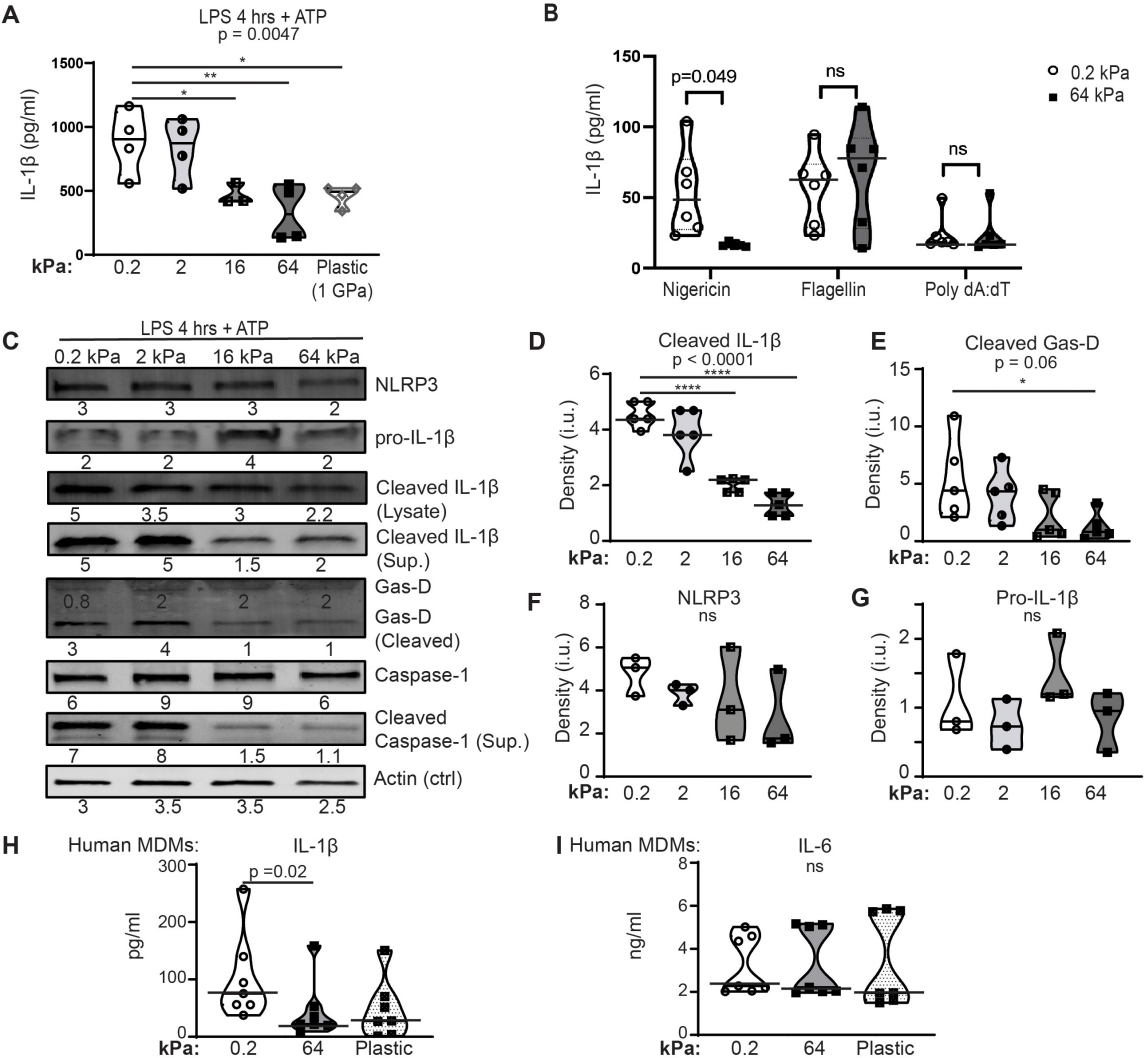
Incubation on stiffer substrates reduces NLRP3-mediated IL-1β production. **(A)** IL-1β quantified by ELISA from supernatants of BMDMs cultured on collagen-coated silica gels of indicated stiffness or plastic. BMDMs were primed with LPS (4 h) then activated with ATP (30 min). **(B)** IL-1β quantified by ELISA from supernatants of BMDMs cultured on collagen-coated silica gels, primed with LPS, then exposed to nigericin (NLRP3 activator), flagellin (NLRC4 activator), or poly dA:dT (AIM2 activator). **(A, B)** Each symbol represents value from one sample, line at median, data combined from three independent experiments, p-value determined using Mann-Whitney. **(C)** Immunoblot of indicated proteins in cell lysates from BMDMs incubated on collagen-coated silica gels of the indicated stiffness and stimulated with LPS (4 h) and ATP (30 min). Densitometric analysis given below corresponding bands. Full immunoblot shown in **Supplementary Figure 6**. **(D-G)** Densitometry from immunoblots of **(D)** cleaved IL-1β (cell lysates), **(E)** cleaved Gasdermin-D, **(F)** total NLRP3, and **(G)** full- length pro- IL-1β protein. Protein levels normalized to actin expression. Each symbol represents value from independent experiment (3 independent experiments), line at median, values compared by ANOVA with follow-up pairwise testing. *p < 0.05, ****p<0.0001 **(H, I) (H)** IL-1β or **(I)** IL-6 in supernatants quantified by ELISA from HMDMs incubated on collagen-coated silica gels of indicated stiffness or plastic, primed with LPS x 4 h then activated with ATP for 30 min. Each symbol represents value from one sample, solid line indicates median, p-value determined using Mann-Whitney (0.2 v 64 kPa). Data combined from two independent experiments.

Induction of IL-1β release via activation of the NLRP3 inflammasome is associated with macrophage-mediated host defense. We previously showed that activation of the NLRP3 inflammasome is mechanosensitive in primary murine alveolar macrophages (AMs), with increased production of the pro-inflammatory cytokine IL-1β induced by incubation on softer substrates (16). To test if NLRP3 mechanosensitivity is generalizable to other macrophage lineages, we measured IL-1β production after NLRP3 priming and assembly in BMDMs (**Figure 6A**). NLRP3-induced IL-1β production requires two separate steps *in vitro*. Priming, induced by LPS or other TLR agonists, activates NF-κB and induces upregulation of the NLRP3 receptor and pro-IL-1β. IL-6 and TNFα are also induced by NF-κB activation. A second stimulant, such as ATP or nigericin, induces rapid polymerization (assembly) of NLRP3, the adaptor ASC, and pro-caspase-1 into the active inflammasome. Assembly of NLRP3 activates caspase-1, which then cleaves pro-IL-1β into bioactive IL-1β. Activated caspase-1 also cleaves gasdermin-D. Cleaved gasdermin-D creates pores in the cell membrane, resulting in cell death (pyroptosis) and release of IL-1β. We use induction of NLRP3, pro-IL-β, IL-6 and/or TNFα as measures of priming. We use detection of cleavage products of IL-β, gasdermin-D, or caspase-1 as measures of assembly.

NLRP3-mediated IL-1β production was mechanosensitive in BMDMs (**Figure 6A**). BMDMs incubated upon substrates of 0.2 and 2 kPa (soft) produced more IL-1β than did BMDMs incubated on substrates of 16 or 64 kPa, or upon plastic (stiff). In contrast, IL-1β production induced by NLRC4 or AIM2 inflammasomes (activated by exposure to flagellin or to poly dA:dT, respectively) was not mechanosensitive (**Figure 6B**; **Supplementary Figure 3**). Analysis of NLRP3 components, substrates and cleavage products by immunoblot further confirmed prior observations that NLRP3 assembly, but not priming, is mechanosensitive (**Figures 6C–G**; **Supplementary Figure 4**). Cell lysates from BMDMs incubated on collagen-coated silica gels and stimulated with LPS + ATP were probed for NLRP3 receptor, gasdermin D, caspase-1, and pro-IL-1β to assess priming. Lysates were also probed for cleaved gasdermin D, caspase-1, IL-1β to measure assembly (**Figure 6C**; **Supplementary Figure 4**). Consistent with mechanoregulation of NLRP3 assembly, levels of cleaved IL-1β (**Figure 6D**) and cleaved gasdermin-D (**Figure 6E**) were reduced on stiffer substrates. Equivalent expression of the NLRP3 receptor (**Figure 6F**) and of pro-IL-1β (**Figure 6G**) are consistent with intact priming, as previously observed in AMs (16).

Mechanoregulation of NLRP3-induced IL-1β production was also tested in human monocyte- derived macrophages (HMDMs) (**Figures 6H, I**). HMDMs cultured on collagen-coated silica gels of 0.2 or 64 kPa, or cultured on plastic, were primed with LPS and activated with ATP. As seen in murine AMs and BMDMs, IL-1β production was reduced on stiffer substrates (**Figure 6H**), but IL-6 production was equivalent ( (16); **Figure 6I**). Priming is sufficient to induce IL-6, while IL-1β production requires both priming and assembly. Equivalent IL-6 production suggests that LPS priming is not regulated by substrate stiffness, while reduced IL-1β is consistent with mechanoregulation of NLRP3 assembly. Thus, we find that mechanoregulation of NLRP3-mediated IL-1β production occurs in murine AMs, murine BMDMs, and human MDMs, indicating a regulatory mechanism generalizable to multiple macrophage lineages.

### 3.5 LA1 recapitulates mechanoregulation of NLRP3 assembly

**Figure 7:**
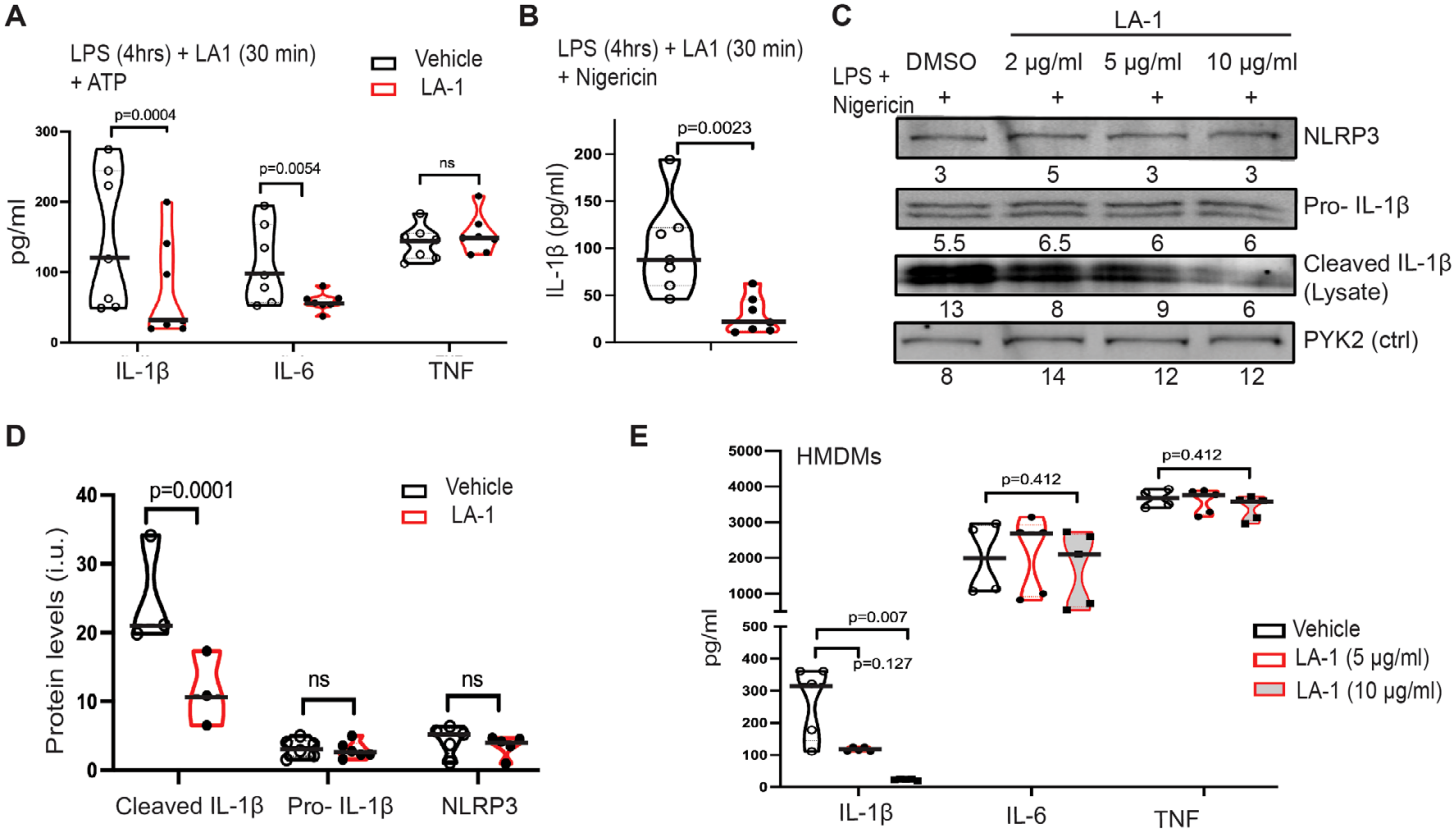
LA1 treatment mimics NLRP3 downregulation by stiff substrate. **(A)** Production of IL-1β, IL-6, and TNFα from BMDMs primed with LPS (4 h) then activated with ATP (30 min), with or without LA-1 (2 µg/ml or vehicle control) for final 30 min of priming and during ATP activation. Concentrations of cytokines in cellular supernatant was determined by ELISA **(B)** IL-1β production from BMDMs cultured with (red) or without (grey) LA1 (2 µg/ml) during final 30 min of LPS priming and activation with nigericin (30 min). Concentrations determined by ELISA from cellular supernatants. **(A, B)** Each symbol represents value from one of six independent experiments, line at median, p-values determined by Mann-Whitney. **(C)** Immunoblot for indicated proteins in lysates of BMDMs treated with or without LA1 (concentrations as indicated) during final 30 min of LPS priming (4 h) and subsequent activation with nigericin (30 min). Densitometry of each band shown below. Whole immunoblots with molecular weight markers shown in **Supplementary Figure 6**. **(D)** Densitometry of immunoblots for indicated proteins (as in **(C)**), normalized to actin. Each symbol represents value from one of at least three independent biologically independent experiments, line at median, p-values determined by Mann-Whitney. **(E)** Production of IL-1β, IL-6, and TNFα from HMDMs primed with LPS (4 h) and treated with or without LA-1 (5 or 10 µg/ml) for the final 30 min of LPS priming and during activation with ATP (30 min). Cytokine concentrations in cellular supernatant determined by ELISA. Each symbol represents value from one sample, line at median, p-values determined by Mann-Whitney or **(H)** ANOVA. Data combined from two independent experiments.

We next determined if LA1-mediated activation of CD11b would mimic incubation on stiff substrates by downregulating NLRP3 activation (**Figure 7**). We added LA1 to BMDMs during the last half hour of the four-hour priming with LPS, then activated cells with ATP (**Figure 7A**). LA1 exposure reduced IL-1β and IL-6 production, but not that of TNFα. IL-1β production was also reduced by LA1 stimulation when nigericin was used to activate NLRP3 (**Figure 7B**). We tested three concentrations of LA1, finding that LA1 concentrations of 2 and 10 µg/ml, but not 0.5 µg/ml, was sufficient to downregulate IL-1β and IL-6 production (**Supplementary Figure 5A, B**). While NLRC4-mediated production of IL-1β was not regulated by substrate stiffness, LA1 treatment exhibited a trend (p=0.06) towards reducing NLRC4-mediated IL-1β. LA1 exposure had no effect on AIM2-mediated IL-1β production (**Supplementary Figure 5C**).

Immunoblot of cell lysates was again used to assess upregulation of NLRP3 receptor and pro-IL-1β during LPS-mediated priming and of cleavage of pro-IL-1β after activation (**Figure 7C**; **Supplementary Figure 6**). While NLRP3 receptor and pro-IL-1β were expressed equivalently at all concentrations of LA1 tested, cleavage of IL-1β was reduced in a dose-dependent manner (i.e. increasing LA1 concentrations led to decreasing IL-1β cleavage; **Figure 7C**). Levels of cleaved IL-1β, NLRP3 and pro-IL-1β measured by multiple immunoblots confirm that LA1 stimulation did not alter NLRP3 or pro-IL-1β levels, but did reduce production of cleaved IL-1β (**Figure 7D**). Finally, we found that LA1 treatment also reduced NLRP3-mediated IL-1β production, but not that of IL-6 or TNFα, in human MDMs (**Figure 7E**). Thus, treatment of two macrophage lineages with the CD11b agonist, LA1, was sufficient to mimic the effect of stiff substrate on NLRP3-mediated IL-1β production. We summarize our comparison of LA1 exposure to incubation on stiff and soft substrates in **Table 1**.

**Table 1:** Summary of results directly comparing effects of substrate stiffness to LA1 treatment during macrophage activation and/or polarization.

Outcome	Substrate stiffness	LA1 exposure
Differentiation	Yes; stiffer➔ increased	No effect observed
Proliferation	No effect observed	No effect observed
TNFa after LPS	No effect observed	No effect observed
IL-6 after LPS	No effect observed	LA1➔Reduced
iNOS	Softer➔ increased	Not done
Surface markers	CD86, MHCII, ICAM-1, CD74, CD206, CD36, CD209a, CD31 are mechanosensitive CD40, CD9 not mechanosensitive	CD86, CD40, CD74, CD206, CD36, CD209a, CD31 regulated by LA1 MHCII, ICAM-1, CD9 not regulated by LA1
NLRP3-mediated IL-1β	Stiffer➔ reduced	LA1➔ reduced
NLRC4-mediated IL-1β	No effect observed	Trends (p = 0.06), possible reduction by LA1
AIM2-mediated IL-1β	No effect observed	No effect observed

LA1 treatment mimics the effect of incubating on stiffer substrates by downregulating NLRP3-mediated IL-1β production. However, LA1 exerts significantly different results on surface marker expression and IL-6 production, compared to stiffer substrates.

### 3.6 Interrogation of signaling molecules downstream of LA1 exposure

**Figure 8:**
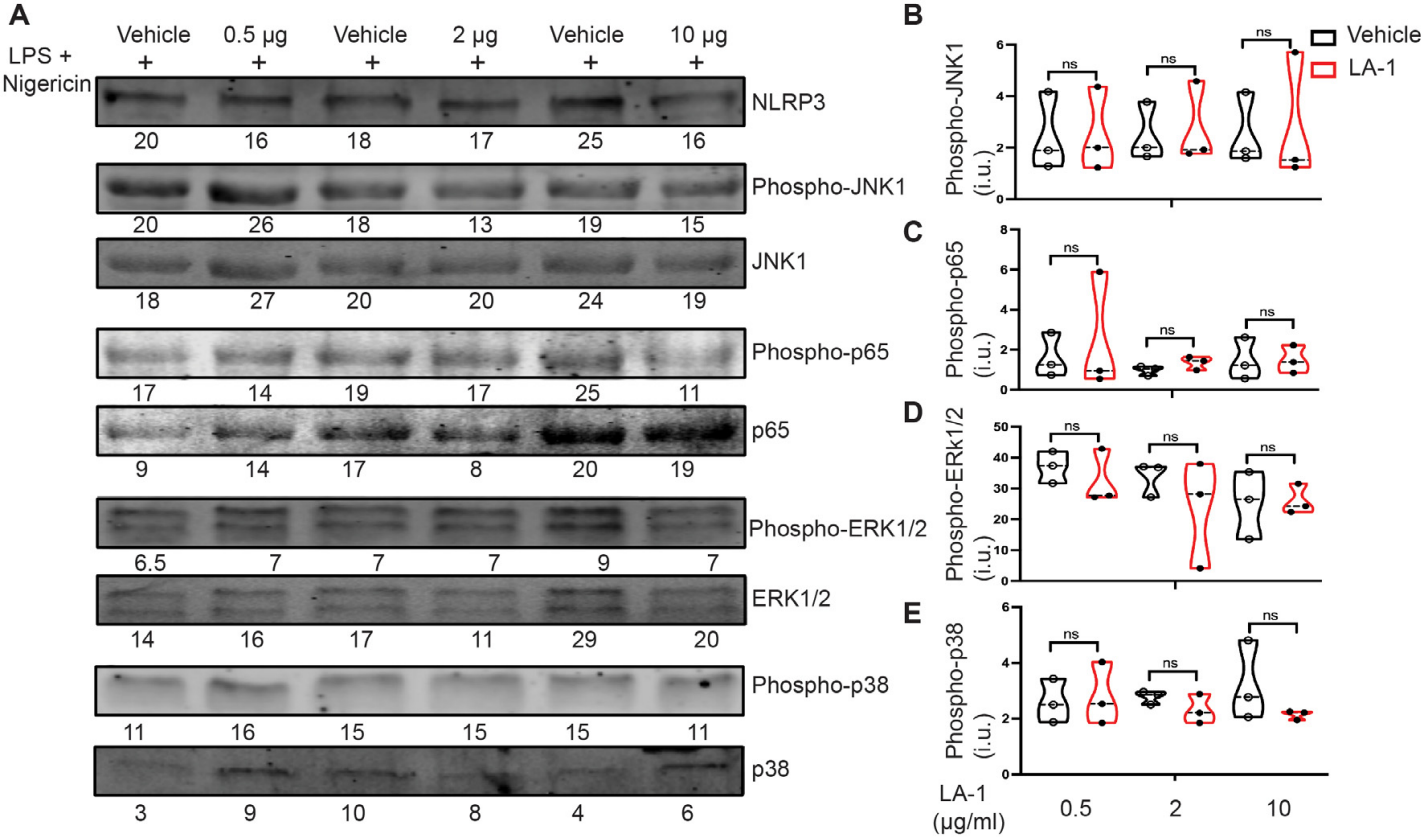
Interrogation of candidate tyrosine kinases downstream of LA1-mediated CD11b activation. **(A)** Immunoblot of lysates derived from BMDMs incubated with or without LA1 during final 30 min of LPS priming (4 h) and activation with nigericin (30 min). Densitometry of probed proteins shown below corresponding bands. **(B–E)** Quantification of **(B)** phospho-JNK1, **(C)** phospho-p65 (NF-κB) **(D)** phospho-ERK1/2, and **(E)** phospho-p38 normalized to total substrate (JNK1, p65, ERK1/2, or p38, respectively), then actin. Each symbol represents value from one of the 3 independent experiments, line at median, p-values determined using Mann-Whitney. Whole immunoblots with molecular weight markers shown in **Supplementary Figure 7**.

Multiple signaling pathways are activated downstream of integrin ligation, including the MAP kinases ERK-1/2, p38 and JNK (34, 35). Activation of the transcription factor NF-κB induces pro-inflammatory signals associated with host defense, such as IL-6 and TNFα (36, 37). To determine which, if any, of these pathways was modulated by treatment with LA1, we probed lysates of BMDMs incubated with varying concentrations of LA1 during the final 30 min of LPS priming and nigericin-mediated activation for phospho-ERK-1/2, phospho-p65, phospho-p38 and phospho-JNK (**Figure 8A**, **Supplementary Figure 7**). Total NLRP3, ERK-1/2, p65 (NF-κB), p38 and JNK were used as loading controls. We found no differences in phosphorylation of JNK1 (**Figure 8B**), p65 (**Figure 8C**), ERK-1/2 (**Figure 8D**) or p38 (**Figure 8E**) at any concentration of LA1 tested. Thus, we do not find evidence that LA1-mediated activation of CD11b transduces signals through MAP kinases or p65.

**Figure 9:**
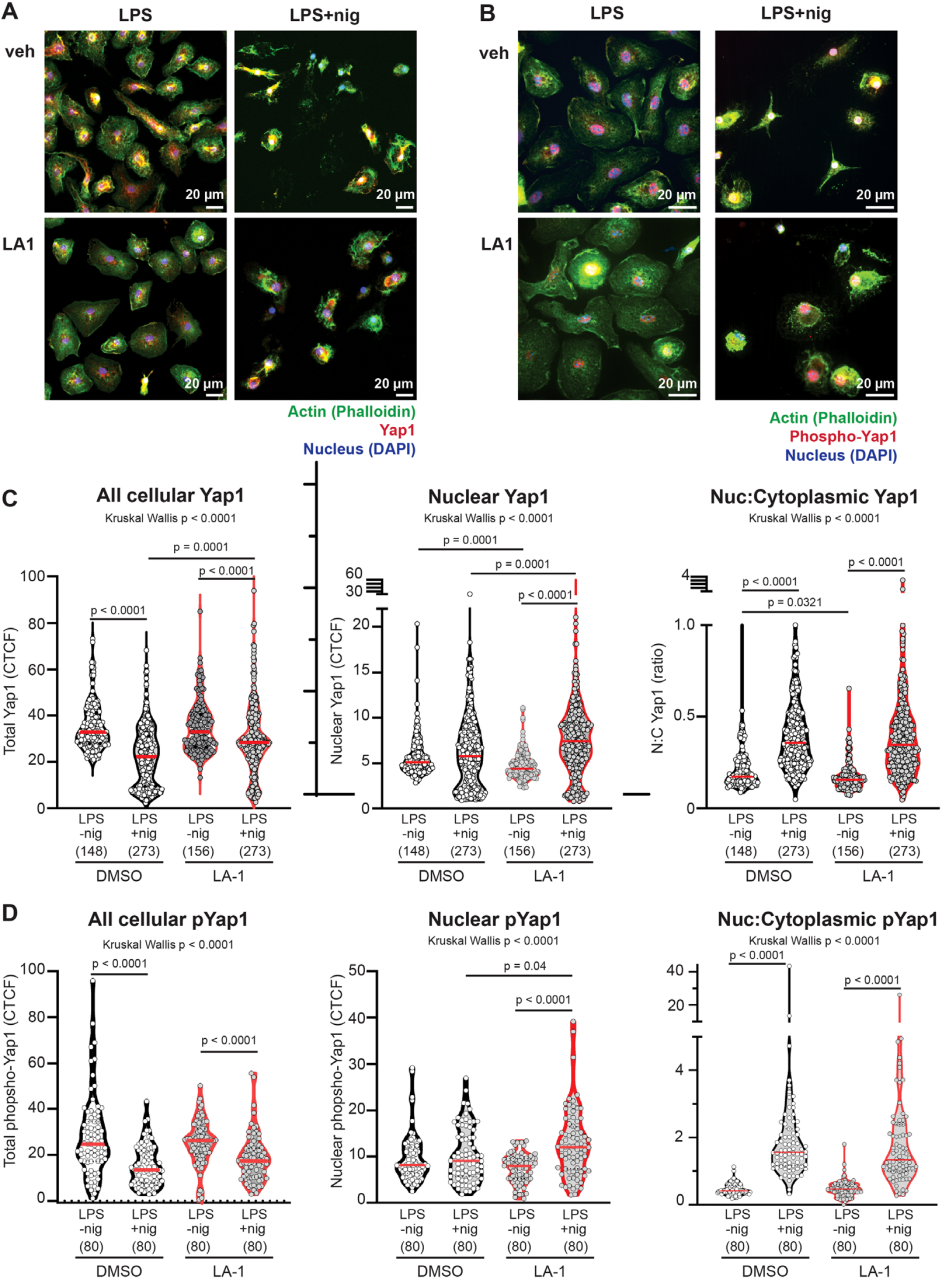
**(A, B)** Confocal images of BMDMs primed with LPS, treated with or without LA1 (2 ug/ml) for final 30 min of priming, then activated with nigericin (30 min). Cells were fixed, permeabilized and labeled with phalloidin to illuminate actin (green), DAPI to illuminate nuclei, and **(A)** anti-Yap1 or **(B)** anti-phospho (S127) Yap (red). Colocalization of green and red signals result in yellow. An example of one image separated into red, green and blue channels, and an explanation of methodology for quantification of localization, are provided in **Supplementary Figure 8**. Scale bar = 20 µm. Yap1 or P-S127-Yap1 localization was quantified by intensity (corrected total cell fluorescence; CTCF) of each cell or nucleus. Cytoplasmic CTCF was determined by using the following CTCF = Integrated Density – (Area of selected cell X Mean fluorescence of background readings). **(C, D)** CTCF of total cellular, nuclear, or ratio of nuclear:cytoplasmic CTCF of **(C)** Yap or **(D)** phosphorylated-Yap1. Each symbol represents value from one cell, solid line shows the median. P values were determined by Kruskal-Wallis with follow-up tests for multiple comparisons. Data combined from three independent experiments, with n=total cells for each condition shown below x-axis.

Mechanotransduction has also been associated with phosphorylation, degradation, and nuclear translocation of the transcription factor Yap1 (28, 29, 38). The most commonly recognized pathway governing Yap1 phosphorylation and nuclear translocation is the Hippo pathway, in which phosphorylation of Yap at Ser127 results in sequestration by 14-3-3 in the cytoplasm (39). Furthermore, Ser127 phosphorylation of Yap has been reported to reduce total Yap1 expression and suppress NLRP3 activation (40). Because of these prior reports showing that Yap1 is mechanotransducing, and that Yap1 can modulate NLRP3 activation, we tested if LA1 modified Yap1 phosphorylation and/or localization. We were unable to detect endogenous total Yap1 expression or phospho-Yap1 by immunoblot in BMDMs in our system, presumably due to low protein expression. We therefore analyzed localization of Yap1 and phospho-Yap1 by confocal microscopy (**Figures 9A, B**). Because we are analyzing via confocal, we were unable to clearly separate phospho-Yap1 and Yap1 in the same cells. Therefore, we are not able to report the ratio of phospho-Yap1 to Yap1 on a per cell basis. Cellular and nuclear Yap1 and pYap1 were quantified using corrected total cell fluorescence (CTCF).

In untreated BMDMs, NLRP3 activation with LPS+nigericin induced significant reductions in total cellular Yap1 and phosphoYap1 (**Figures 9C, D**), compared to BMDMs that were primed with LPS. However, the amount of Yap1 or phosphoYap1 that colocalized with the nucleus was unchanged after NLRP3 activation with nigericin. The calculated ratio nuclear to cytoplasmic Yap1 or pYap1 was therefore significantly increased by nigericin in untreated cells (**Figures 9C, D**).

Nuclear localization of Yap1 was significantly reduced in cells primed with LPS and exposed to LA1 (median ratio 0.15, range 0.07-0.65) compared to untreated cells primed with LPS (median 0.17, range 0.09-1.6). In other words, there was greater cytoplasmic localization of Yap1 in LPS-primed cells treated with LA1 (**Figure 9C**). There was no difference in phospho-Yap in LPS-primed cells exposed to LA1, compared to untreated LPS-primed cells (**Figure 9D**). After NLRP3 activation with nigericin, cells exposed to LA1 also showed significant decreases in total cellular Yap1 and pYap1. However, the amount of total Yap1 in LA-1 treated cells was significantly higher than in untreated cells after nigericin activation. Additionally, in LA1-treated cells, there was significantly higher nuclear Yap1 and phospho-Yap1 in nigericin-activated cells compared to LPS-primed (**Figures 9C, D**). Nuclear Yap1 and nuclear pYap1 localization was significantly increased in LA1-treated cells activated by nigericin, compared to untreated cells (**Figure 9C**). Thus, we show that direct stimulation of the integrin α-chain CD11b with a small molecule increased Yap1 phosphorylation and reduced NLRP3-mediated production of IL-1β.

## 4 Discussion

Macrophages reside in all tissues of the body and maintain homeostasis through interactions with other cells and through secretion of cytokines (26, 30, 41, 42). When challenged by invading pathogens, macrophages adopt a microbicidal, pro-inflammatory phenotype to promote host defense. *In vitro*, the pro-inflammatory phenotype has been modeled by exposure to IFNγ + LPS, and termed “classically-activated” or “M1.” After tissue injury or clearance of infection, macrophages shift to a pro-healing phenotype, modeled *in vitro* by exposure to IL-4 and IL-13, and termed “alternatively-activated” or “M2.” While the M1/M2 dichotomy is useful shorthand, the binary division of phenotypes oversimplifies the profound plasticity of macrophages, which can simultaneously support host-defense and tissue repair (2).

Macrophages integrate multiple external signals to sustain homeostasis. Signals include cytokines, chemokines, exposure to pathogen- or damage-associated microbial patterns (PAMPs/DAMPs), engagement with surface receptors on neighboring cells, and tissue mechanics (7). Elucidating how cellular mechanics regulate macrophage polarization mechanoregulation must be considered when evaluating macrophage responses, because every tissue-resident macrophage lineage uniquely adapts to its physiological environment (19). Each tissue environment exposes macrophages to varied mechanical signals, such as substrate compliance, stretch or strain (7). For example, Kupffer cells reside within the static and relatively stiff liver environment, while cardiac macrophages are exposed to continuous stretch and relaxation. Tissues such as lung, brain and spleen, are softer than cardiac muscle (19). Furthermore, tissue compliance changes in disease states, with decreased compliance during infection, cancer and fibrosis. In addition to effects on tissue-resident macrophages, mechanical signals may affect proliferation and differentiation of peripheral blood monocytes recruited into tissues to enhance host defense or tissue repair (43–45).

Multiple signaling pathways transduce mechanical signals, including the stretch-activated calcium channel Piezo1 (25, 28, 46) and integrins (9, 19). Integrins are heterodimeric transmembrane ligands that transmit mechanical information through interactions with the actin cytoskeleton (7, 9, 47–49). The integrin CD11b/CD18, also called Mac-1 or CR3, is highly expressed on monocytes and macrophages and engages multiple extracellular matrix components, including collagen (50). Recently, a first-in-class integrin α-chain activator, LA1, was tested for efficacy as an anti-tumor agent in a phase 1/2 clinical trial (15). Unfortunately, the trial was terminated early due to lack of evidence for efficacy. LA1 is a small molecule that binds and activates CD11b, inducing a conformational change from the “bent, closed” low-affinity to the “extended, open” high-affinity state. LA1 was developed as an anti-inflammatory agent, intended to increase CD11b-mediated adhesion and tissue retention, and thereby impair trafficking of inflammatory monocytes and neutrophils to sites of inflammation (51). LA1 has been shown in experimental (pre-clinical) models to improve survival of kidney allografts (52), to prevent primary graft dysfunction in lung transplants (53), and to protect mice against endotoxin-induced shock (13). LA1 was hypothesized to accelerate tumor clearance by reducing accumulation of myeloid-derived suppressor cells and tumor-associated macrophages into solid tumors (15). However, LA1 administration showed no effect on tumor clearance in human trials.

One explanation for the failure of LA1 in clinical trials, despite promising outcomes in pre-clinical models, would be the inhibition (or activation) of additional CD11b- mediated inflammatory processes not yet recognized. Because integrins support mechanotransduction as well as trafficking, and because mechanical cues are known to regulate different aspects of macrophage activation and polarization, we examined the mechanosensitivity and LA1-sensitivity of the biological processes employed to classify macrophage polarization: differentiation, proliferation, expression of surface markers, cytokines, and NLRP3 inflammasome activity. As detailed below, LA1 treatment mimicked some, but not all, effects of incubation on stiffer substrates. We did identify multiple effects of LA1 treatment, including reduction of NLRP3 activation, IL-6 production, and alteration in surface marker expression levels, that should be considered when repurposing LA1 for additional clinical uses.

We first analyzed proliferation and differentiation of bone marrow-derived monocytes into macrophages *in vitro*, using the standard protocol of one week exposure to M-CSF, while incubating cells on collagen-coated silica gels to model varying tissue compliance. We selected gel compliances representing best estimates of soft tissues, such as healthy lung (0.2 kPa) and of diseased tissue, such as fibrotic lung (64 kPa, stiff) (54). Studies of other cell types have shown that soft substrates enhance proliferation of mesenchymal stem cells (55), while stiffer substrates promote macrophage proliferation (56). Substrate stiffness can also alter the terminal differentiation choices of multiple stem cell lineages (20, 57, 58). We found that incubation on stiff substrate significantly accelerated monocyte to macrophage differentiation. We did not detect mechanosensitivity of monocyte or macrophage proliferation. Proliferation of monocytes *in vitro* supports recent findings that under certain inflammatory conditions, monocytes can undergo rapid proliferation to fill a niche (23, 24). We also did not find any effect of single dose LA1 treatment on macrophage proliferation or differentiation. In contrast, analysis of proliferation of the murine cell line Raw 264.7 in 3-dimensonal agarose gels suggested that stiffer environments may reduce proliferation (59). Clarifying when, which and how mechanical cues control macrophage differentiation and proliferation will greatly aid in understanding the profound heterogeneity of monocyte and macrophage populations found in disease states such as idiopathic pulmonary fibrosis.

Our results examining cytokine production challenge the prevailing model suggesting stiffness promotes pro-inflammatory signaling (7, 19, 28). We found no mechanosensitivity of IL-6 cytokine production when BMDMs were incubated on a range of compliances (0.2-64 kPa) and exposed to LPS, nor did we see any mechanosensitivity of TNFα protein production. The only mechanosensitive cytokine we detected was IL-10, with incubation on 0.2 kPa gels resulting in higher IL-10 concentrations after exposure to IFNγ+LPS. In contrast to prior reports (60), we also found higher expression of iNOS when cells were incubated on 0.2 kPa gels compared to 64 kPa. Our system differs from this prior report in several important aspects. First, our range of compliances tested uses a “soft” gel of 0.2 kPa compared to a “stiff” gel of 64 kPa; in other studies, 11 kPa or < 50 kPa was considered “soft,” with compliances of > 100 kPa defined as “stiff.” (7, 19, 28, 36). In fact, our results indicating no effect of substrate stiffness on IL-6 or TNFa production for stiffnesses of 0.2 – 64 kPa align with prior results, in which inhibition of TNFa and IL-6 was only observed at a stiffness of 230 kPa (36). Other differences include our use of murine BMDMs, compared to use of myeloid cell lines, and our use of collagen-1 to coat the gels, compared to fibrinogen, RGD peptide or decellularized cardiac extracellular matrix (7, 19, 29, 60). These profound differences in technique likely explain our contrasting results, and underline the need for further, rigorous comparison of mechanosensitivity of multiple primary macrophage lineages on multiple ECM components, at physiological compliances.

In contrast to our results on stiff substrates, incubation of BMDMs with LA1 reduced IL-6 production in response to LPS. This result is consistent with prior findings that LA1 pre-treatment reduced IL-6 and TNFα production from human monocytes stimulated with TLR7/8 or TLR2 agonists (R848 and Pam3csk4, respectively) (61). A difference between LA1 treatment and incubation on stiffer substrates could be due to a variety of explanations, including that integrins other than CD11b/CD18 are mediating mechanotransduction in our BMDMs, or that direct agonism of CD11b by LA1 engages more CD11b/CD18 integrins that incubation on collagen-coated surfaces. Further investigation will be required to distinguish between these possibilities.

In addition to cytokine secretion, macrophage polarization has been classified by the up- or downregulation of various surface markers, including CD86, MHC class II, ICAM-1, CD40, and CD74 (associated with host defense), and upregulation of CD9 and CD206 (associated with tissue repair) (30). However, in our system, the analysis of surface marker expression on macrophages incubated on soft or stiff substrate and exposed to either IFNγ+LPS or IL-4+IL-13 defied binary classification. We analyzed our data using a two-way ANOVA, to detect if mechanosensation and/or cytokine stimulation had independent or interacting effects on expression levels of the indicated surface markers. Most markers were significantly regulated by both mechanotransduction and by chemokine stimulation, but the “directionality” of changes in markers induced by soft vs. stiff substrate did not parallel changes induced by cytokine exposure. For instance, IFNγ+LPS upregulated CD86, CD74 and CD40, all of which have been previously reported to be associated with a “host defense” phenotype (30). However, CD86 is significantly upregulated on soft substrate, CD74 is most upregulated on stiff substrate, and CD40 expression is unaffected by changes in substrate compliance. Downregulation of CD9 by IFNγ+LPS and upregulation by IL-4+IL-13 is consistent with prior reports that CD9 is associated with the tissue repair phenotype (30) – but both IFNγ+LPS downregulation and IL-4+IL-13 upregulation are enhanced by incubation on soft substrate. A new paradigm for describing the plasticity of macrophage phenotypes is needed to capture the intricacy and complexity of macrophage integration of environmental cues (2).

Comparison of LA1 exposure to substrate stiffness as an integrin activator revealed additional complexity. In general, surface markers showed less dependence upon LA1 treatment than upon substrate stiffness. LA1 treatment and incubation upon stiff substrates similarly regulated CD36, CD209a and CD31 showed similar responsiveness. Interestingly, CD40 was modulated by LA1 but not by substrate stiffness. While most markers exhibited significant regulation by mechanical cues or integrin ligation it is not possible to neatly categorize changes in markers into binary subsets. Our data underscore that the binary division of macrophage polarization into M1 and M2 is too simplistic to capture macrophage plasticity. Additionally, it is incorrect to view soft or stiff substrates as consistently “pro-inflammatory” or “pro-healing,” because mechanotransduced signals are integrated with additional external signals. Changes in expression markers reflect the profound plasticity of macrophages in integrating and adapting to multiple environmental cues.

Finally, we analyzed mechanotransduction to NLRP3 inflammasome assembly. While we previously found that the actin-binding protein L-plastin (LPL) mediated macrophage mechanotransduction, localization of the kinase Pyk2, and activation of NLRP3, we exclusively analyzed primary murine AMs (16). We now show that NLRP3 assembly and/or NLRP3-mediated production of IL-1β is mechanosensitive in murine BMDMs and in human MDMs. NLRP3 mechanosensitivity is thus generalizable across multiple macrophage lineages and is relevant to human primary cells. We confirmed that IL-1β production is increased when macrophages are incubated on softer substrate, further challenging the prevailing theory that stiffer substrates promote inflammation. Furthermore, we reveal that LA1 treatment of cells last 30 min of priming had no apparent effect on the expression of NLRP3, pro-IL-1β or TNFα, but did significantly reduce NLRP3-mediated release of mature IL-1β from BMDMs and MDMs. Our results are concordant with a prior publication that studied macrophage inflammatory signaling across a physiological range (0.6 kPa – 100 kPa) of compliances (62).

The finding that LA1 treatment reduces NLRP3 assembly, similarly to incubation on stiff substrates, enables additional mechanistic analyses. For instance, obtaining high-quality confocal microscopy images of cells incubated on gels is technically challenging, because it is difficult to image through the silica gel to the cell surface engaged with the gel surface. Confocal imaging of cells treated with LA1 is, however, straightforward. We therefore tested downstream signaling pathways induced by LA1 engagement of CD11b. We found no evidence that LA1 ligation altered NF-κB or the MAP kinases ERK1/2, JNK, or p38 downstream of NLRP3 activation. However, LA1 ligation did significantly alter the expression and localization of Yap1 and phospho-Yap1. In untreated cells, NLRP3 activation was accompanied by significant loss of total cellular Yap1. Reduced NLRP3 activation after LA1 treatment correlated with a smaller loss of cellular Yap1. LA1 treatment also induced higher nuclear levels of Yap1 and phopho-Yap1.

At first glance, our results showing that reduced Yap1 levels correlate with increased NLRP3 activation, appear contradictory to prior reports, in which diminished Yap1 was associated with reduced NLRP3 activation (40). However, there are significant differences in the experimental systems, and Yap1 regulation is highly complex (63). In the previously reported model, Yap1 in the cell cytoplasm directly bound to NLRP3, preventing ubiquitination and degradation of NLRP3. When Yap1 expression was reduced through knock-down (40), NLRP3 was exposed to ubiquitination and degradation. Phosphorylation of cytoplasmic Yap1 also targets Yap1 for degradation, resulting in subsequent loss of NLRP3. Diminished levels of NLRP3 result in reduced levels of IL-1β after activation. Notably, these studies focused on the effect of Yap1:NLRP3 cytoplasmic interactions prior to NLRP3 activation (40). In our study of endogenous Yap1 after NLRP3 activation, we find that nuclear localization of Yap1 is rapidly increased, although total cellular Yap1 and pYap1 are significantly reduced. Although phosphorylation of Yap1 is often considered to retain Yap1 in the cytoplasm, phospho-Yap1 can translocate to the nucleus when actomyosin contractility is abrogated (64). It is possible that the rapid loss of Yap1 after NLRP3 activation correlates with “release” of NLRP3 from Yap1, with subsequent polymerization of the NLRP3 and degradation/translocation of Yap1. This possible model would reconcile our observations with the prior report (40). We note that our results here confirm and extend a previous publication examining the effect of LA1 on NLRP3 activation (65). Our current study was not designed to assess if changes in Yap1/phospho-Yap1 expression and localization are upstream, downstream, or independent of effects on NLRP3 assembly. However, the proposed model offers an avenue to explore in future work.

In summary, our results clearly demonstrate that substrate stiffness and direct integrin agonism modulate macrophage polarization towards phenotypes associated with host defense and/or tissue repair. LA1-mediated activation of CD11b mimicked incubation on stiffer substrates in that it inhibited NLRP3 assembly and co-regulated expression of CD86, CD36, CD209a and CD31with cytokine stimulation. LA1 treatment differed from incubation on stiffer substrates in downregulating IL-6 production and possibly reducing NLRC4-mediated IL-1β production (p = 0.06). Differences between results obtained when cells were incubated upon stiffer substrates and when cells were treated with LA1 could be due to engagement of integrins other than CD11b/CD18 by collagen-coated stiff substrates, to unexpected off-target effects of LA1, or to the relatively short-term nature of single dose treatment with LA1 compared to continuous incubation on stiff substrates. Furthermore, effects of LA1 might vary depending on different culture conditions. For instance, serum used in standard cell culture medium contains a variety of integrin ligands, such as fibronectin and laminin, which could obscure LA1 effects (66). Furthermore, effects of LA1 treatment might be enhanced if cells were incubated upon softer substrates, or if substrates were coated with different extracellular matrix proteins. These permutations could be explored in future work. The inhibition of NLRP3-mediated IL-1β production suggests that LA1 could be repurposed to treat NLRP3-mediated inflammatory diseases, such as lung fibrosis (16). The additional effects of LA1 treatment on macrophage polarization should be considered if LA1 is used as a clinical anti-inflammatory agent.

## Data Availability

The raw data supporting the conclusions of this article will be made available by the authors, without undue reservation.
