# Supplementary material for: Mechanosensitivity of macrophage polarization: comparing small molecule leukadherin-1 to substrate stiffness
Source: Front Immunol. 2025 Mar 6;16:1420325. doi: 10.3389/fimmu.2025.1420325 (PMC11922956; doi:10.3389/fimmu.2025.1420325)
Supplement: Supplementary file 1 [file DataSheet1.pdf]

Supplemental Figures

Supplemental Figure 1

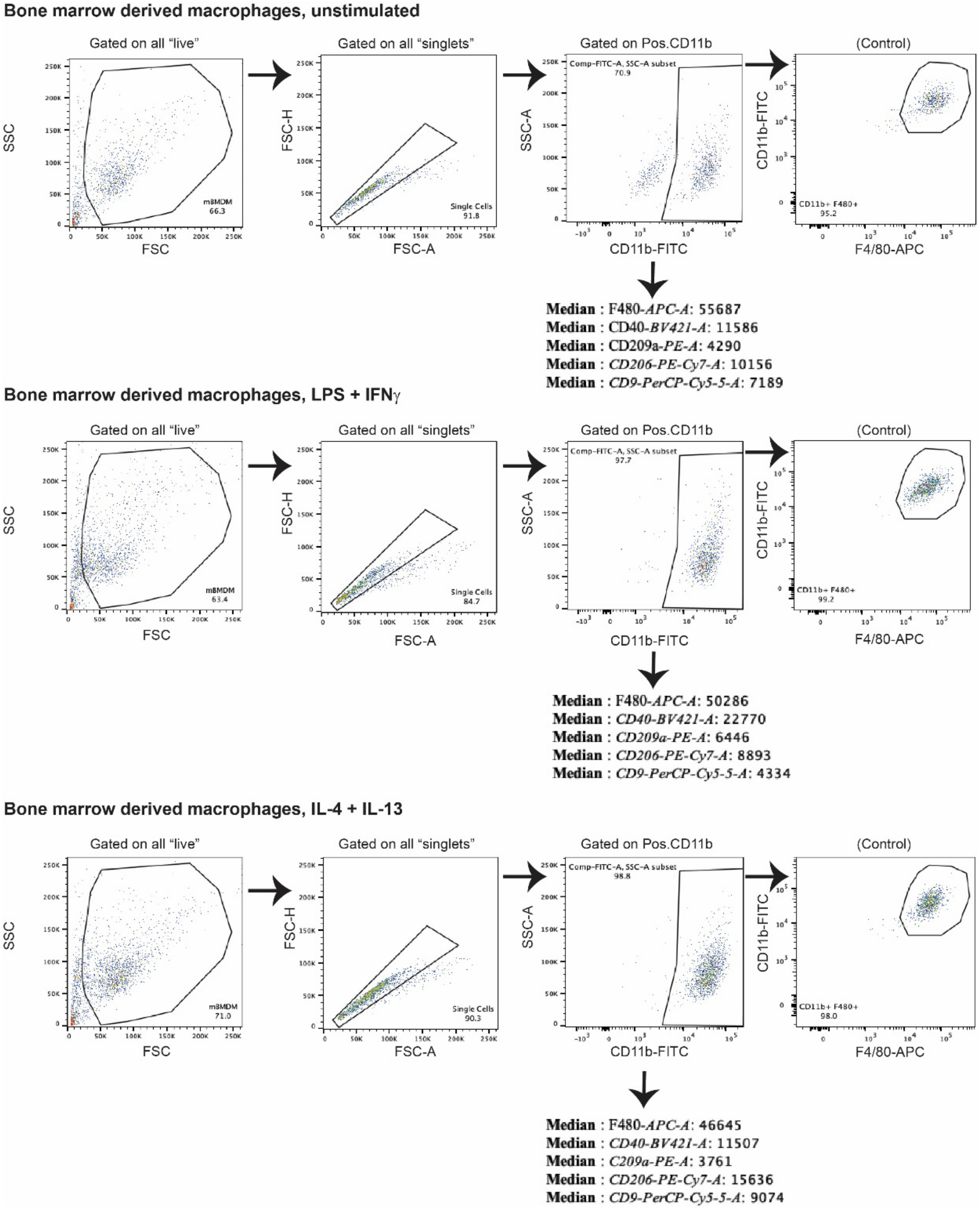

Suppl. Fig. 1. Initial gating strategy for all flow cytometric analyses. Debris and dead cells are excluded by SSC x FSC gating on live cells. Doublets are excluded by gating on singlets (FSC-H x FSC-A). BMDMs are identified by CD11b^{+} and confirmed with F4/80^{high}. Additional gating or quantification of median fluorescence intensity (MFI) per gating strategy shown in manuscript figures.

Supplemental Figure 2

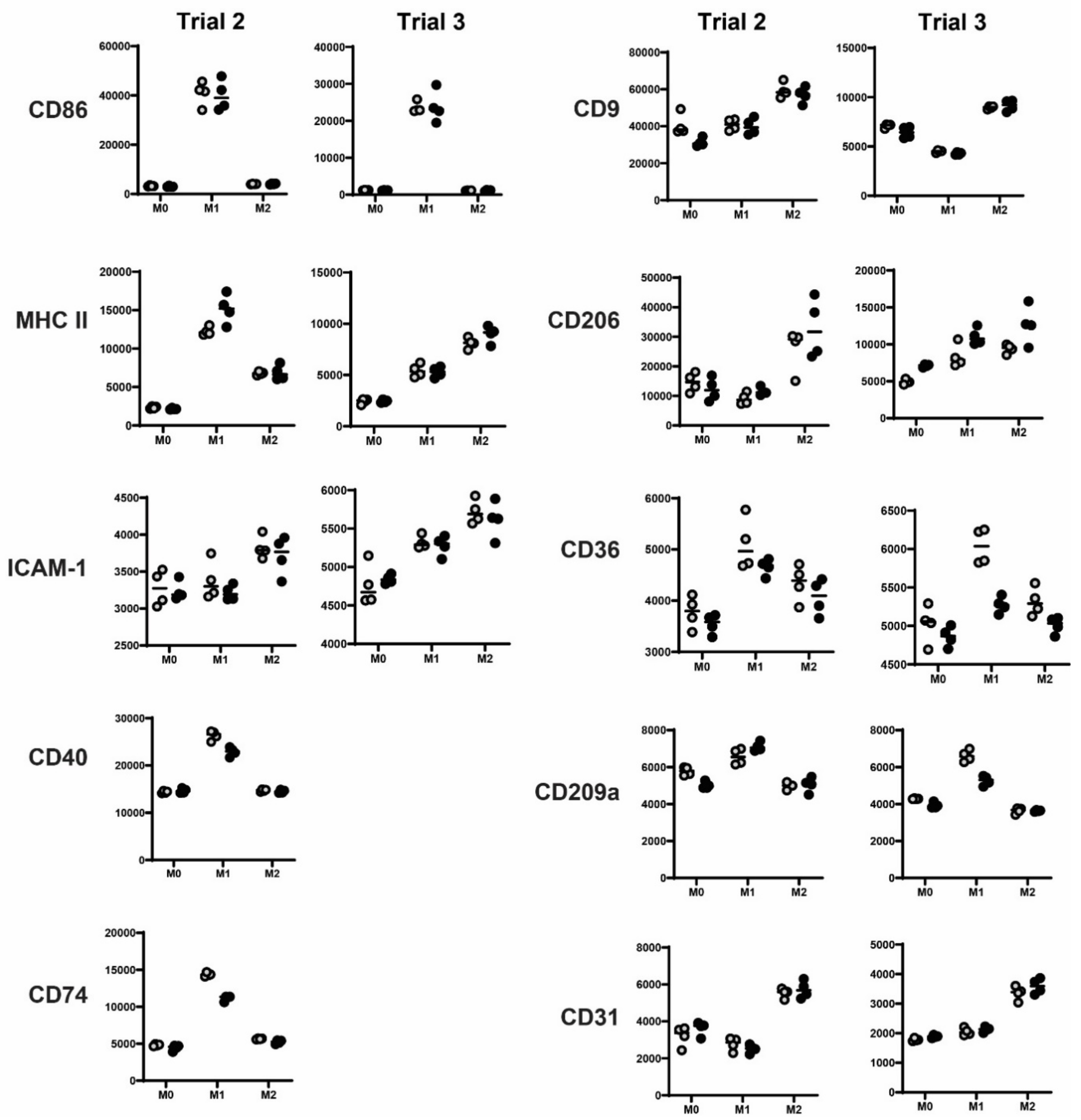

Suppl. Fig. 2. BMDMs were incubated with (closed circles) or without (gray circles) LA1 (5 μg/ml) and with LPS+IFNγ (M1), IL-4+IL-13 (M2), or without specific cytokine stimulation (M0) for 24 h. Expression of indicated surface markers were analyzed by flow cytometry and quantified as mean fluorescence intensity (MFI) of all BMDMs gated ($CD11b^{pos}F4/80^{pos}$). Each symbol represents value from one sample of 4 technical replicates; bar shows median value. Representative experiment with statistical analysis shown in Fig. 5; shown here are results from independent experiments (biological replicates).

Supplemental Figure 3

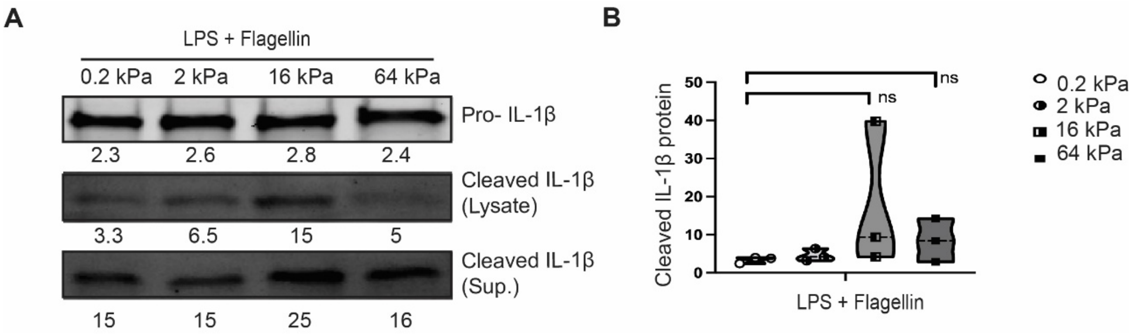

Suppl. Fig 3. (A) Immunoblot of cell lysates from BMDMs incubated on collagen-coated gels of the indicated stiffness, primed with LPS, then exposed to flagellin (NLRC4 activator). Densitometry of probed proteins given below corresponding bands. **(B)** Densitometry of cleaved IL-1β from lysates of BMDMs incubated on substrates of indicated stiffness, primed with LPS and exposed to flagellin. Cleaved IL-1β density normalized to actin (loading control). Each symbol represents value from one of three independent experiments, solid line shows the median. P-values tested using ANOVA test.

Supplemental Figure 4

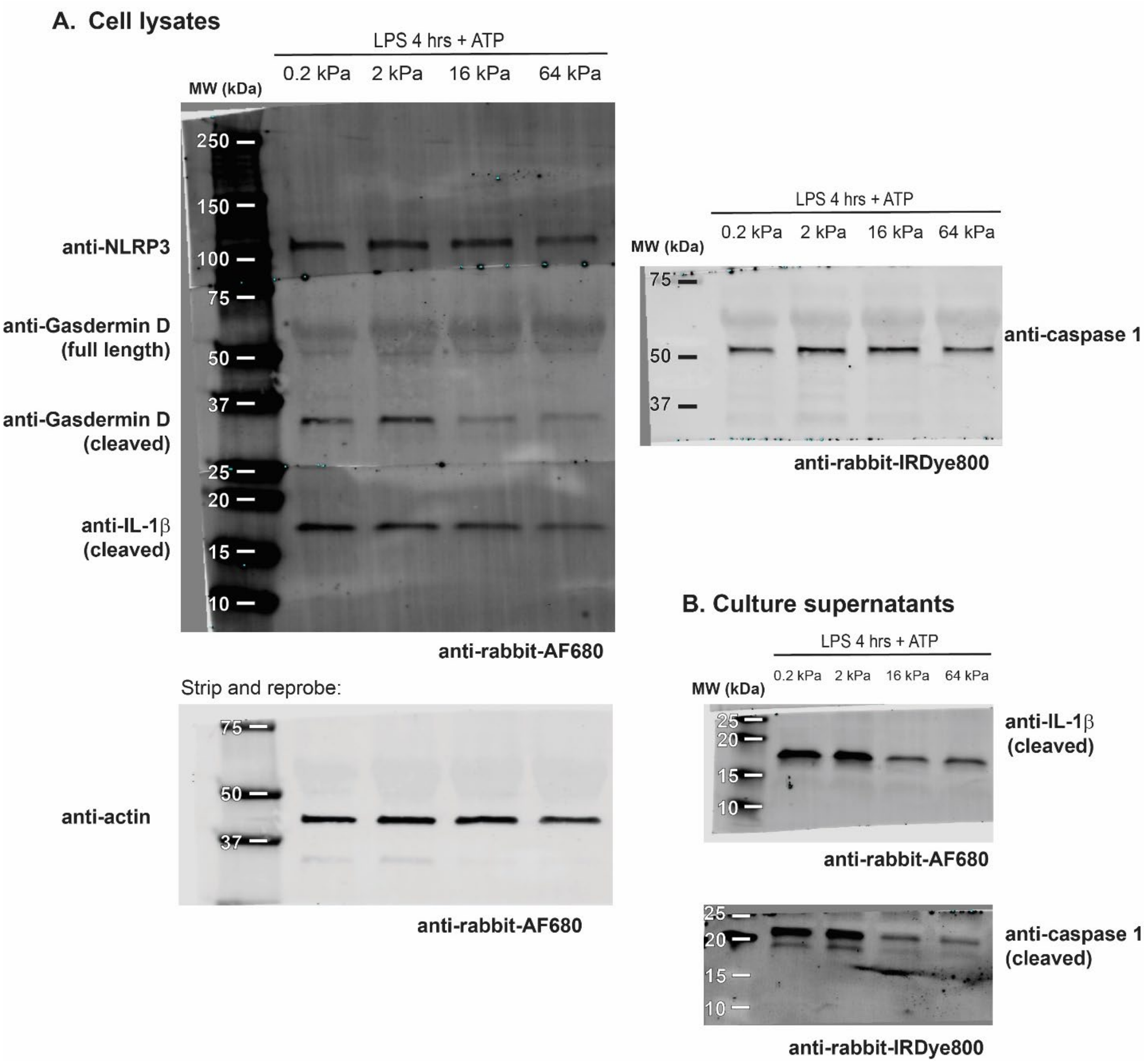

Suppl. Fig. 4. Complete images of immunoblots acquired using LiCOR Odyssey system for cropped images shown in Fig. 6C. (A) Immunoblots for indicated proteins in cell lysates from BMDMs incubated on collagen-coated silica gels of the indicated stiffness and stimulated with LPS (4 h) and ATP (30 min). Samples from each biological samples were divided between two parallel gels to visualize caspase-1 and gasdermin. Upper gel was stripped and reprobed for actin. **(B)** Supernatants were divided between two parallel gels to visualize cleaved IL-1β and cleaved caspase-1.

Supplemental Figure 5

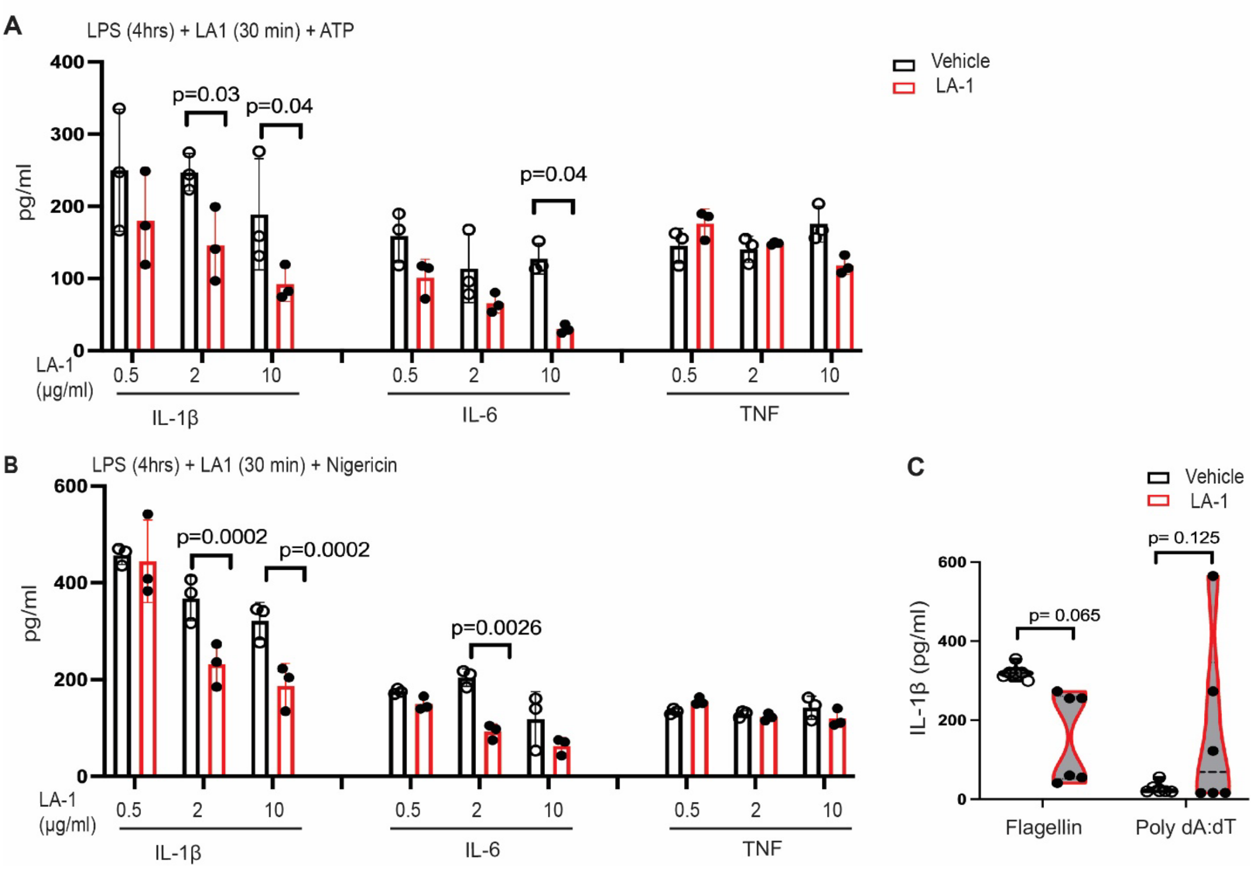

Suppl. Fig. 5. LA1 reduces NLRP3-mediated IL-1β production. (A, B) IL-1β, IL-6 or TNFα was quantified by ELISA from supernatants of BMDMs primed with LPS (4 h) with addition of LA-1 (0.5, 2 or 10 µg/ml) for final 30 m of priming. NLRP3 was activated with (A) ATP or (B) nigericin (30 min). **(C)** IL-1β quantified by ELISA from supernatants of BMDMs primed with LPS (4 h) with addition of vehicle (DMSO) or LA-1 (2 µg/ml) for final 30 m of priming. NLRC4 or AIM2 inflammasomes activated using flagellin or poly dA:dT, respectively. (A,B,C) Each symbol represents an individual data point, bars show median values, and the dashed line shows the interquartile range. Data combined from at least three independent experiments. P-values determined by ANOVA.

Supplemental Figure 6

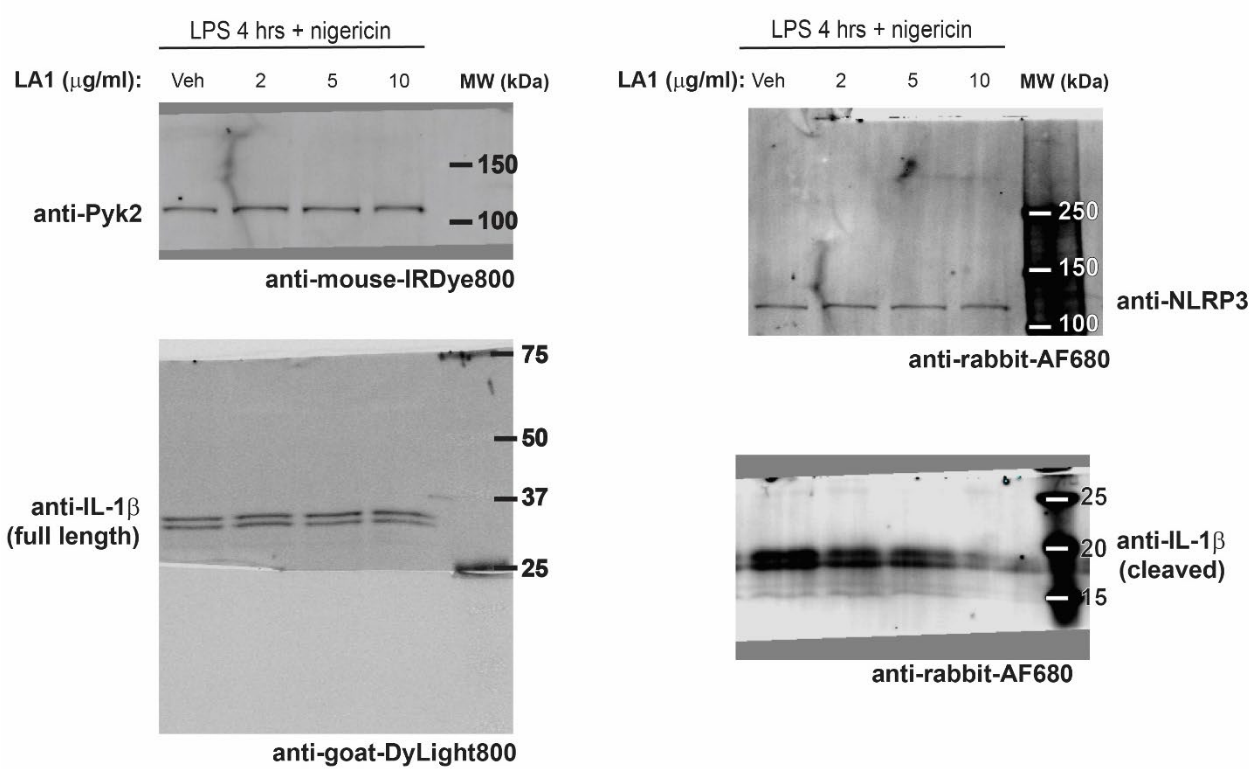

Suppl. Fig. 6. **Complete images of immunoblots acquired using LiCOR Odyssey system for cropped images shown in Fig. 7C.** Immunoblot for indicated proteins in lysates of BMDMs treated with or without LA1 (concentrations as indicated) during final 30 m of LPS priming (4 h) and subsequent activation with nigericin (30 min).

Supplemental Figure 7

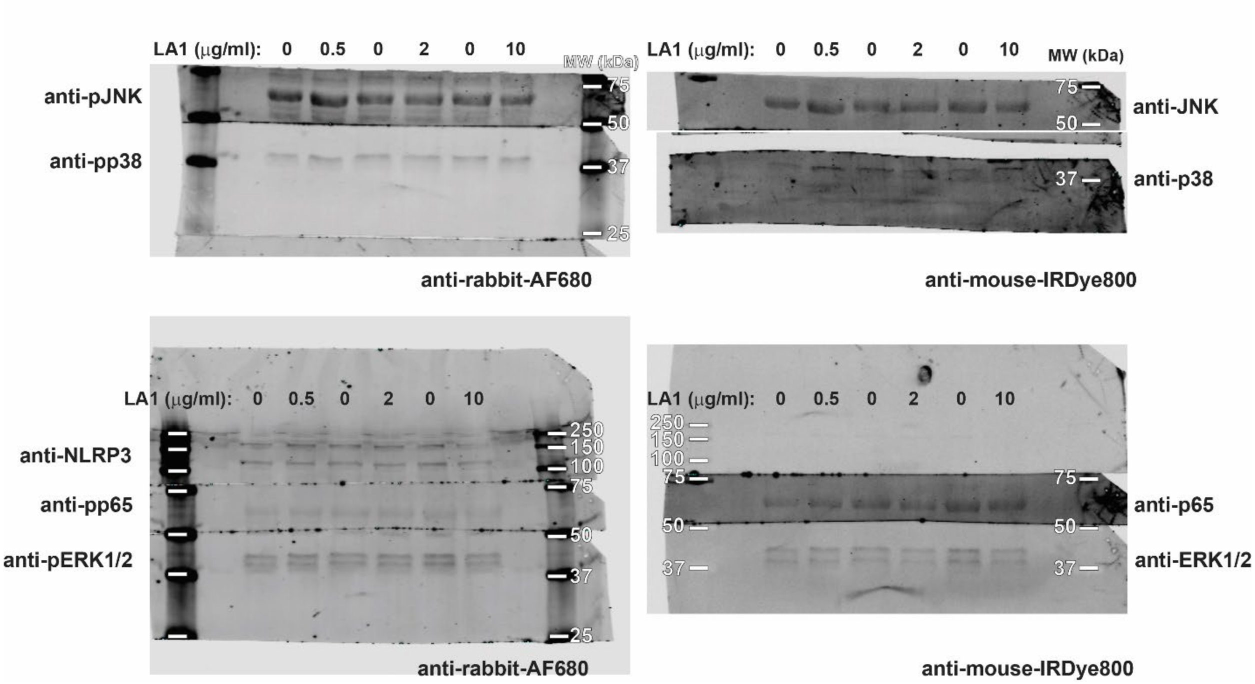

Suppl. Fig. 7. Complete images of immunoblots acquired using LiCOR Odyssey system for cropped images shown in Fig. 8A. Immunoblot of lysates derived from BMDMs incubated with or without LA1 during final 30 m of LPS priming (4 h) and activation with nigericin (30 min).

Supplemental Figure 8

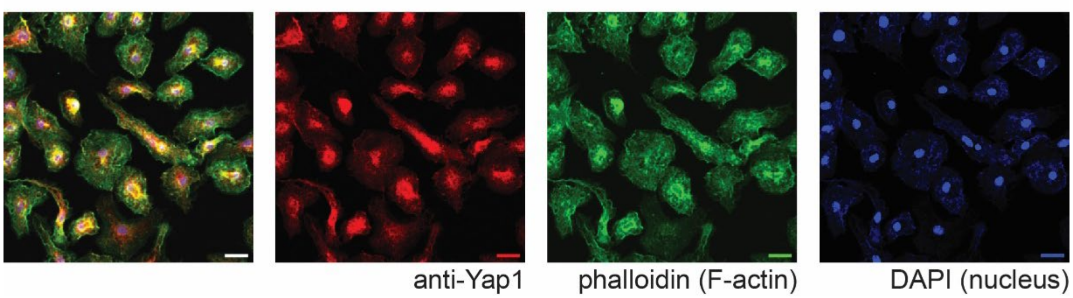

Suppl. Fig. 8. Separation of confocal image by color channel to show individual anti-Yap1, phalloidin, and DAPI staining of WT BMDMs incubated with LPS only. To determine nuclear localization, the DAPI channel was visualized in Image J/FIJI and the nucleus manually circumscribed. The signal intensity of the anti-Yap1 or anti-pYap1 (red) fluorescence within the circumscribed nuclei were then quantified. Total cell fluorescence was determined by using the green (F-actin) channel to manually circumscribe each cell body. Total red fluorescence signal intensity within the circumscribed cell border was then quantified. Scale bar = 20 µm.
